# Unsupervised Characterization of Temporal Dataset Shifts as an Early Indicator of AI Performance Variations: Evaluation Study Using the Medical Information Mart for Intensive Care-IV Dataset

**DOI:** 10.2196/78309

**Published:** 2025-12-03

**Authors:** David Fernández-Narro, Pablo Ferri, Alba Gutiérrez-Sacristán, Juan M García-Gómez, Carlos Sáez

**Affiliations:** 1 Biomedical Data Science Lab Instituto Universitario de Tecnologías de la Información y Comunicaciones Universitat Politècnica de València Valencia, Valencia Spain; 2 Department of Biomedical Informatics Harvard Medical School Harvard University Boston, MA United States

**Keywords:** AI, artificial intelligence, data quality, machine learning, temporal variability

## Abstract

**Background:**

Reusing long-term data from electronic health records is essential for training reliable and effective health artificial intelligence (AI). However, intrinsic changes in health data distributions over time—known as dataset shifts, which include concept, covariate, and prior shifts—can compromise model performance, leading to model obsolescence and inaccurate decisions.

**Objective:**

In this study, we investigate whether unsupervised, model-agnostic characterization of temporal dataset shifts using data distribution analyses through Information Geometric Temporal (IGT) projections is an early indicator of potential AI performance variations before model development.

**Methods:**

Using the real-world Medical Information Mart for Intensive Care-IV (MIMIC-IV) electronic health record database, encompassing data from over 40,000 patients from 2008 to 2019, we characterized its inherent dataset shift patterns through an unsupervised approach using IGT projections and data temporal heatmaps. We trained and evaluated annually a set of random forests and gradient boosting models to predict in-hospital mortality. To assess the impact of shifts on model performance, we checked the association between the temporal clusters found in both IGT projections and the intertime embedding of model performances using the Fisher exact test.

**Results:**

Our results demonstrate a significant relationship between the unsupervised temporal shift patterns, specifically covariate and concept shifts, identified using the IGT projection method and the performance of the random forest and gradient boosting models (*P*<.05). We identified 2 primary temporal clusters that correspond to the periods before and after *ICD-10* (*International Statistical Classification of Diseases, Tenth Revision*) implementation. The transition from *ICD-9* (*International Classification of Diseases, Ninth Revision*) to *ICD-10* was a major source of dataset shift, associated with a performance degradation.

**Conclusions:**

Unsupervised, model-agnostic characterization of temporal shifts via IGT projections can serve as a proactive monitoring tool to anticipate performance shifts in clinical AI models. By incorporating early shift detection into the development pipeline, we can enhance decision-making during the training and maintenance of these models. This approach paves the way for more robust, trustworthy, and self-adapting AI systems in health care.

## Introduction

Leveraging trustworthy artificial intelligence (AI) and machine learning (ML) in health critically depends on reusing data from diverse sources collected over extended periods. However, the inherent variability in medical data and their acquisition processes often results in significant shifts in data distributions [1-3]. At the population level, data-generating processes are generally nonstationary, leading to shifting statistical patterns over time. These shifts can arise from changes in clinical protocols, environmental or seasonal variations, inherent biological and social behaviors, or unexpected biases caused by systematic or random errors [4,5]. Such variability between training and test datasets, whether temporal or site-specific, can severely undermine the development and performance of ML models in health [6-10]. These variations in data distributions are classified as dataset shifts [11,12] and are especially relevant in a nonstationary environment, such as real-world health data [2,13]. Addressing dataset shifts proactively is crucial to ensure the reliability and effectiveness of the training and prospective continual use of health AI.

In classification problems involving input (x) and output (y) variables, a dataset shift occurs when the joint probability distribution of the data differs between training and test datasets. This can happen, for instance, when translating a model to a different site or during the AI’s use or adaptation over time (equation 1).



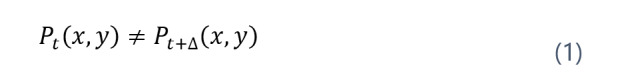



In this work, we considered 3 primary sources of dataset shift: covariate shift, prior probability shift, and concept shift [11], as described next.

Covariate shifts refer to changes in the marginal features distribution over time, such as changes in the distribution of the population (equation 2) [14].



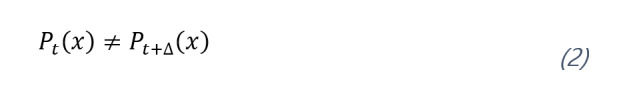



Prior probability shifts refer to changes in the distribution of each class p(y) over time (equation 3) [15].



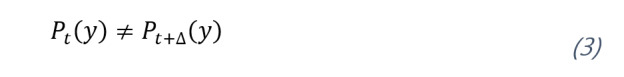



Concept shifts refer to changes in the posterior distribution p(y|x) [11] over time (equation 4).



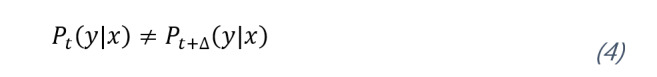



Approaches to identifying dataset shifts can be broadly classified into supervised and unsupervised methods. Supervised methods, such as progressive error and external holdout [16], require evaluation of the target model to detect shifts. Progressive error involves tracking the performance of the model over time to identify deviations, while external retention evaluates the model’s performance on a held-out dataset collected over different periods [11]. Additionally, domain adaptation methods like transfer component analysis and domain-adversarial neural networks help in adapting models to new domains by aligning feature representations across different distributions [17,18]. These techniques can be effective but are often model-dependent and require labeled data, limiting their applicability and flexibility.

On the other hand, unsupervised methods do not rely on model performance and can be applied directly to the data. Techniques such as computing statistical distances (eg, Kullback-Leibler divergence, Wasserstein distance) between distributions, novelty detectors, and discriminative distance measures are commonly used [4,16,19]. Change detection algorithms, such as the Page-Hinkley test and the cumulative sum algorithm, detect changes in the statistical properties of data streams in real time, making them particularly useful for online monitoring of data shifts [20]. Reconstruction-based methods, such as autoencoders, detect shifts by measuring reconstruction error on new data, where high reconstruction error can indicate a distribution shift [21].

Among these unsupervised methods, the Information Geometric Temporal (IGT) projection proposed by Sáez et al [4] provides an unsupervised approach to characterization and exploratory visualization of distributional changes over time. The IGT methodology estimates the statistical distributions of data over time and projects their temporal evolution through nonparametric statistical manifolds. This method presents a robust, straightforward, and model-agnostic way to characterize dataset shifts. It excels in its ability to capture the dynamic nature of data distributions, making it highly sensitive to subtle changes that other techniques might miss. Furthermore, its nonparametric nature ensures flexibility and applicability across various datasets and AI models, enhancing its utility in diverse health settings.

In this study, we investigate how temporal changes in the Medical Information Mart for Intensive Care-IV (MIMIC-IV) health record repository [22] influence the performance of AI models, aiming to develop more reliable and robust health AI applications. Through early, unsupervised characterization of dataset shifts, we seek to identify early indicators that can predict performance variations before they impact clinical outcomes. To detect these shifts, we used the IGT projection method implemented in the *EHRTemporalVariability* package using R software (version 1.2.1; R Foundation for Statistical Computing) [3,4], previously validated in health care data studies [3,23,24]. The combination of temporal heatmaps and IGT projections provided actionable insights into the underlying dynamics of the data. By training AI models in annual data batches, we were able to link these temporal shifts with changes in performance metrics. Our findings demonstrate the potential of early characterization of temporal dataset shifts as a proactive monitoring tool to anticipate performance shifts over time. This proactive identification of dataset shifts paves the way for the development of reliable AI systems, leading to improved health outcomes and increased reliability in clinical settings.

## Methods

### Overview

**Figure 1:**
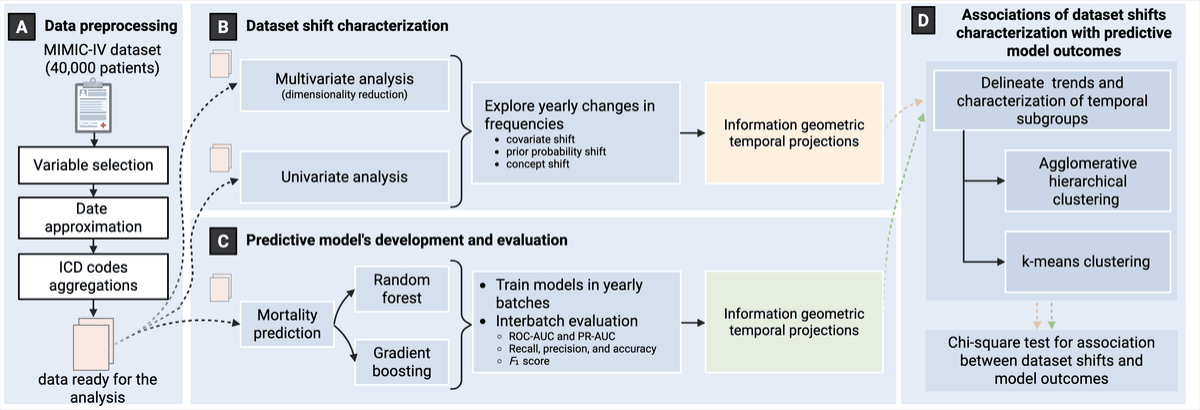
Workflow of the study phases to assess the relationship between the temporal dataset shift characterization of the Medical Information Mart for Intensive Care-IV (MIMIC-IV) database and the machine learning models developed to predict the mortality of the patients. (A) Data preprocessing steps. (B) Dataset shift characterization. (C) Predictive model’s development and evaluation. (D) Association of dataset shifts characterization with predictive models outcome process. ICD: International Classification of Diseases; PR-AUC: precision recall area under the curve; ROC-AUC: receiver operating characteristic area under the curve.

Our study comprised 4 phases, including the MIMIC-IV data preparation, the characterization of temporal dataset shifts, the characterization of changes in predictive models’ performance metrics, and the test for association between the dataset shifts and model performance metrics (Figure 1). Next, we describe each of the phases of the methodology.

### MIMIC-IV Dataset

The MIMIC-IV dataset contains electronic health records (EHRs) of more than 40,000 patients from the Beth Israel Deaconess Medical Center (BIDMC) [22]. The MIMIC-IV dataset contains information sourced from the intensive care unit, which is known for its abundance of data and its use in other AI-related works [9]. This modern EHR dataset spans a decade of admissions from 2008 to 2019 and includes precise digital records, such as the electronic medicine administration record. The data are organized modularly, allowing for easy linking to external departments.

For our aim of in-hospital mortality prediction, we included adult patients (age ≥18 years) with at least one hospital admission in the MIMIC-IV dataset. The input features include, on one hand, characteristics of each patient, such as the gender, age, or ethnic group, and on the other hand, the *ICD* (*International Classification of Diseases*) codes of the diagnoses and procedures of each admission in the versions 9 (*ICD-9* [*International Classification of Diseases, Ninth Revision*], from 2008 to 2015) and 10 (*ICD-10* [*International Statistical Classification of Diseases, Tenth Revision*], from 2016 to 2019). Each admission can be linked with multiple *ICD* codes for procedures and diagnoses.

### Data Preprocessing

Since we aim to evaluate dataset shifts and model performance over time, the temporal identifier of data is of utmost importance. In MIMIC-IV, admission dates are anonymized and grouped into 3-year cohorts spanning 2008-2019. To approximate the admission year for each record, we applied the date preprocessing method developed by Yao et al [9], which assigns each admission to a specific year within its cohort (see Section 1.1 in Multimedia Appendix 1 for details). This process allows for yearly analysis but introduces a possible deviation of up to ±1 year for any given admission. Consequently, some admissions from, for example, 2015 or 2016 may be labeled as 2014.

To reduce the dimensionality of the dataset and the effect that updating the codes could have on temporal variability, all the *ICD-9* and *ICD-10* codes have been grouped and mapped into common chapters [25] using information from the Chronic Condition Indicator from the Healthcare Cost and Utilization Group [26]. Despite the increased number of codes between *ICD-9* (13,000 codes) and *ICD-10* (68,000 codes), both keep a hierarchical structure that allows the aggregation of the codes at higher levels. In both systems, codes are organized into chapters or categories, representing a broader group of diseases. Regarding diagnosis codes, the *ICD-9* codes are aggregated into 19 chapters (Table S1 in Multimedia Appendix 1), while the *ICD-10* codes are aggregated into 21 (Table S2 in Multimedia Appendix 1) [27]. Furthermore, we created some final chapters by merging the corresponding chapters from both *ICD-9* and *ICD-10* versions (Table S3 in Multimedia Appendix 1). Regarding procedure codes, we aggregated them into 18 chapters for the *ICD-9* version (Table S4 in Multimedia Appendix 1) and 17 for the *ICD-10* version (Table S5 in Multimedia Appendix 1). Then, these chapters were merged into 17 final chapters (Table S6 in Multimedia Appendix 1). Most of the *ICD-9* procedure chapters were merged into a single final chapter, Medical and Surgical, linked to only 1 *ICD-10* chapter based on the merging method used. This could reduce the degree of detail in our study. We decided to expand this final chapter into 13 different subchapters to provide a more detailed mapping of the *ICD-9* chapters into the *ICD-10* ones. (Table S7 in Multimedia Appendix 1). Lastly, given the multiplicity in the number of *ICD* codes per patient, the final groups were coded using one-hot encoding to allow a richer, unordered input for the predictive models regarding the *ICD* codes.

### Dataset Shift Characterization

To characterize dataset shifts, we used the unsupervised approach by Sáez et al [3,4]. This method enables an exploratory characterization and quantification of temporal changes using data temporal heatmaps (DTHs) and IGT projections [5]. The DTHs allow users to dynamically explore changes in absolute and relative frequencies of the variables over time and simultaneously at multiple variable values. The IGT projections embed time batches as a series of points in a latent information space, namely a geometric statistical manifold of nonparametric information from the temporal batches. The IGT projection is obtained, first, by calculating the dissimilarity matrix of the probability distribution functions in temporal batches and, second, by embedding the batches through their dissimilarity matrix with multidimensional scaling in a reduced dimensional space of 2 or 3 dimensions. Consequently, the data batches are represented as temporally indexed points in a latent information space where distances between them correspond to the dissimilarity of their statistical distributions. The IGT projection allows for delineating trends as continuously flowing time batches, abrupt changes as gaps between groups of batches, temporal subgroups as clusters of batches, and seasonality as temporal cycles [3]. Additionally, the IGT projection enables the empirical characterization of conceptually related temporal subgroups using unsupervised machine-learning techniques on the projected temporal points.

We characterized covariate and prior probability shifts using the DTHs and IGT projections provided by the *EHRTemporalVariability* package. To capture concept shifts, we extended the library to compute both class-conditional feature distributions p(x|y) and posterior probabilities p(y|x) and then visualized their temporal evolution with corresponding DTHs and IGT plots.

To explore temporal relationships and patterns in both categorical (eg, *ICD* codes) and numerical variables (eg, age), we conducted a multivariate analysis. This approach allowed us to examine how variables evolve collectively over time, revealing trends, cycles, or structural shifts in the data. We applied factorial analysis of mixed data (FAMD) [28], a dimensionality reduction technique suitable for datasets with mixed data types. Unlike t-distributed Stochastic Neighbor Embedding or Uniform Manifold Approximation and Projection (UMAP), FAMD is a linear technique that preserves the global relationships and variance, which aids with the interpretability [29]. We retained the first 3 FAMD dimensions, which together explained 15.16% of the total variance, striking a balance between meaningful representation for our exploratory analysis and computational efficiency. This reduced space enabled clearer visualization of latent patterns while minimizing the computational load, especially for resource-intensive steps like kernel density estimation (KDE), which scales poorly with higher dimensionality. Finally, to determine whether the low variance retained by the first 3 components of the FAMD was adequate for capturing temporal variability, we conducted the same unsupervised characterization analysis using UMAP as the dimensionality reduction method.

Then, we separated the FAMD coordinates into 2 datasets, one for deceased patients and one for nondeceased patients. This allowed us to study how concepts might change over time using conditional probability p(x|y). We derived probability distributions from the FAMD dimensions to obtain the DTHs, which helped us spot shifts in probabilities linked to covariate or concept shifts. Next, we applied a KDE by year on the first 3 FAMD dimensions for the full and conditioned datasets. Then, for the conditioned datasets, we combined their KDE probability maps into a single map. This map was renormalized to calculate the IGT projection for conditional probabilities, using the *EHRTemporalVariability* package [3]. This step helped us identify shifts in concepts and temporal trends. Finally, we calculated the IGT for the entire FAMD dataset to examine covariate shifts and broader temporal trends.

Next, we conducted a univariate analysis to pinpoint which features drive temporal variability. We began by estimating each feature’s marginal distribution in the overall dataset, p(x), to detect covariate shifts, and the class prior, p(y), to detect prior probability shifts. We then estimated the class-conditional feature distributions, p(x|y), for each outcome. Applying Bayes’ theorem (equation 5),



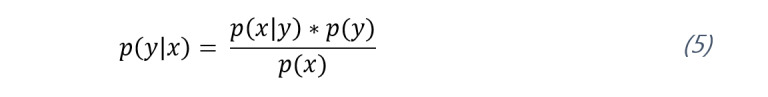



we derived the posterior probability p(y|x) for each feature, thereby isolating concept shifts. Finally, we embedded each of these univariate distributions into an IGT projection. This IGT projection for each distribution allowed us to visualize each feature’s temporal trajectory and systematically identify covariate, prior, and concept shifts over time.

### Predictive Models’ Development and Evaluation

The models developed to predict the in-hospital mortality of the patients in the dataset were based on ensemble learning methods, including random forest and gradient boosting. These methods are at the state of the art for the classification of tabular data [30,31]. Random forest is an ensemble learning method that builds multiple decision trees during training and then combines their predictions to improve overall accuracy and reduce overfitting. Gradient boosting is an ensemble learning technique that iteratively combines weak predictive models, typically decision trees, to create a robust predictive model. Through sequential optimization of an objective function, this method progressively reduces errors, demonstrating significant efficacy in diverse classification and regression tasks within high-dimensional data spaces.

To analyze the performance of the models over time, the initial dataset was batched on a yearly basis from 2008 to 2019. Thus, we used 12 subsets to train and validate the yearly models. We divided each subset following a criterion of 80% for training and 20% for testing. Of the 80% of the training data, 70% was for pure training, and the remaining 30% was used for validation.

We trained each model on the training set and tuned hyperparameters using grid search on the validation set. For random forest and gradient boosting, we predefined values for the number of trees and maximum depth. To reduce computation time, we ran a grid search in parallel. We validated models on data from the same year as training, using the receiver operating characteristic area under the curve (ROC-AUC) as the metric for its robustness to class imbalance and threshold independence [32]. Finally, we averaged ROC-AUC scores across 12 models for each hyperparameter set and selected the configuration with the highest mean score.

Once the hyperparameters were defined, we studied each model’s performance over time, evaluating them on the test sets from all 12 temporal batches. The metrics used to evaluate a more detailed performance of the models, in this case, included the ROC-AUC, precision recall area under the curve, recall (or sensitivity) of the positive class, precision, accuracy, and macro *F*_1_-score. However, due to the high class imbalance (Figure S2 in Multimedia Appendix 1), we used a prediction threshold other than the default 0.5. To determine this threshold, we ranked possible values using the Youden index (equation 6) and selected the one with the highest score using the validation data from the most recent temporal batch. We then applied this optimal threshold to binarize the test data predictions.



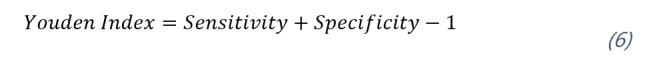



### Association of Dataset Shift Characterization With Predictive Model Outcomes

To prove our objective of determining whether an early unsupervised exploratory characterization of dataset shifts can explain prospective changes in the performance of predictive models, we compared the patterns from the dataset shift characterization, those in the IGT projections, with equivalent embeddings from the interbatch evaluation of AI performance metrics over time. These embeddings, leading to a characterization of the patterns in AI performance metrics, were calculated by applying multidimensional scaling to the interbatch evaluation matrices evaluated year by year and representing the batches in a 3-dimensional space. This procedure is equivalent to the obtention of the IGT for the predictive models’ metrics.

We applied agglomerative hierarchical clustering to identify temporal clusters in both the IGT projection and interbatch model evaluation embeddings. We aimed to delineate temporal subgroups, thus elucidating patterns of similarity in the data and the behavior of the models over time [5]. This allowed us to identify groups of conceptually related time periods in which probability distributions are similar within a group but dissimilar between groups. We used a complete linkage and the Manhattan distance, which, according to Strauss and Maltitz [33], is less sensitive to outliers. To justify the number of clusters to be performed on each case, we applied the elbow method [34] and the analysis of the dendrogram distances, joined to the results extracted from the exploratory unsupervised characterization.

Additionally, we implemented a k-means clustering process to reinforce the results obtained in the hierarchical clustering process. To do so, we initialized the k-means with 2 centroids in the data of the first and last years to follow the natural temporal trend, as it was the optimal number of clusters obtained in the hierarchical clustering.

Finally, to statistically determine the existence of a significant association between the clusters from the dataset shift characterization and the interbatch model evaluation embeddings, we applied the Fisher exact test with a significance level of .05.

### Ethical Considerations

This study used the MIMIC-IV dataset, which is a publicly available deidentified dataset of EHRs from the BIDMC. The dataset was obtained in accordance with the guidelines set forth by the Massachusetts Institute of Technology and the BIDMC Institutional Review Board. Access to the dataset was granted upon completion of the required Collaborative Institutional Training Initiative program course on data use and privacy for researchers, ensuring compliance with ethical standards for using health data. To ensure patient privacy, all data collected at BIDMC as part of routine clinical care are deidentified and transformed before being made available to researchers who have completed human research training and signed a data use agreement [22]. As the data are fully deidentified and publicly accessible, patient consent was not required.

## Results

### Data Preprocessing Results

The final dataset comprised 61 features and 239,246 admissions, of which 232,276 belonged to nondeceased patients and the remaining 6970 (2.91%) to deceased ones. After batching data on a yearly basis, the average number of patients per year was 35,781, with a standard deviation of 27,610 (Figure S1 in Multimedia Appendix 1 shows the amount of data per year).

### Dataset Shifts Characterization

**Figure 2:**
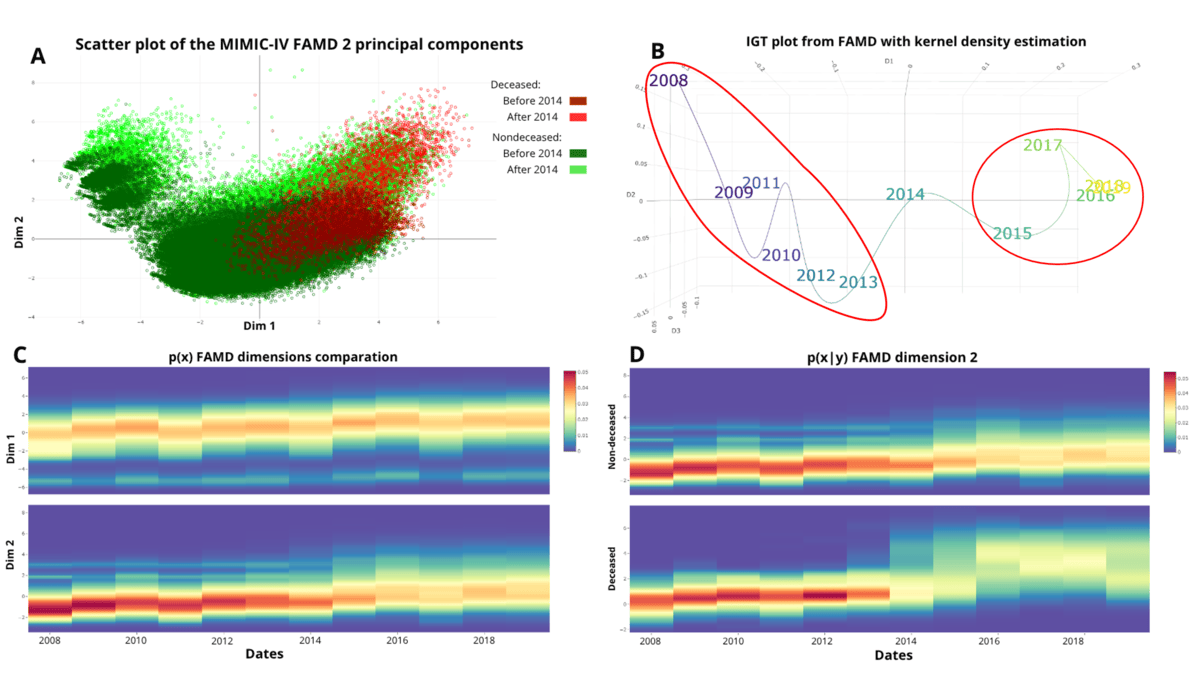
Results of the multivariate dataset shift characterization. (A) Scatter plot of the first 2 dimensions of the studied Medical Information Mart for Intensive Care-IV (MIMIC-IV) dataset’s factorial analysis of mixed data (FAMD). Dim 1 is the largest variance dimension, and Dim 2 is the second largest variance dimension. The data are divided by classes (green and red) and time (darker and lighter colors). (B) Information Geometric Temporal (IGT) projection of the entire dataset after applying the FAMD and the kernel density estimation to the first 3 dimensions. Temporal subgroups are highlighted by red circles. (C) Heatmap of the probability distribution of the first 2 dimensions of the FAMD over time. (D) Heatmap of the conditional probability distribution of each class in the second dimension of the FAMD.

As an overall representation of the data changes over time, Figure 2A shows the first 2 components of the FAMD divided by class and 2 time periods, previous and after 2014, the year the update of *ICD-9* to *ICD-10* took place. These results show that both survivors’ and nonsurvivors’ positions and class-conditional layouts change over time. The nondeceased patients show a wider variance over the first dimension. The magnitude and impact of these changes on AI are further explored.

Figure 2B shows the IGT of the data after applying the FAMD and the KDE to the principal 3 dimensions. The colors in the IGT projection plot represent a gradient that distinguishes different yearly temporal batches. Subsequently, Figures 2C and 2D present temporal heatmaps depicting the temporal variability derived from the first 2 dimensions of the FAMD applied to the entire dataset and the second dimension when segmented by class, respectively. In Figure 2D, we chose the second dimension of the FAMD over the first one because it better represents the presence of the temporal discriminative variability of the classes. The contribution of the variables to the principal components of the FAMD can be examined in Figure S16 in Multimedia Appendix 1. Figure S17 in Multimedia Appendix 1 displays the results of the unsupervised characterization of temporal variability based on the UMAP-reduced data. It is evident that the IGT projection reflects the same trend previously observed with FAMD. Specifically, there are 2 distinct temporal groups, one comprising data from 2008 to 2013 and the other from 2015 to 2019, along with a transition year in between. The rest of the results are obtained for the FAMD dimensionality-reduced data.

A notable increase in the variability of the data’s probability distribution occurs after 2014, coinciding with the adoption of the *ICD-10* coding system, leading to enhanced dispersion across the temporal spectrum. Specifically, 2014, 2015, and 2016 exhibited pronounced shifts in the conditional probability distribution, characterized by significant variations in distribution trends and increased dispersion, which is indicative of concept shifts. Additionally, a distinct covariate shift was detected in 2015, marked by a notable dispersion in data and an ascending trend. The IGT analysis, as depicted in Figure 2, corroborated these phenomena, affirming the initial observations. In the IGT plot, we identified 2 distinguished groups, one from 2009 to 2013 and the other from 2016 to 2019. In the middle, there are some transition years. Thus, the presence of covariate shift and concept shift is justified.

**Figure 3:**
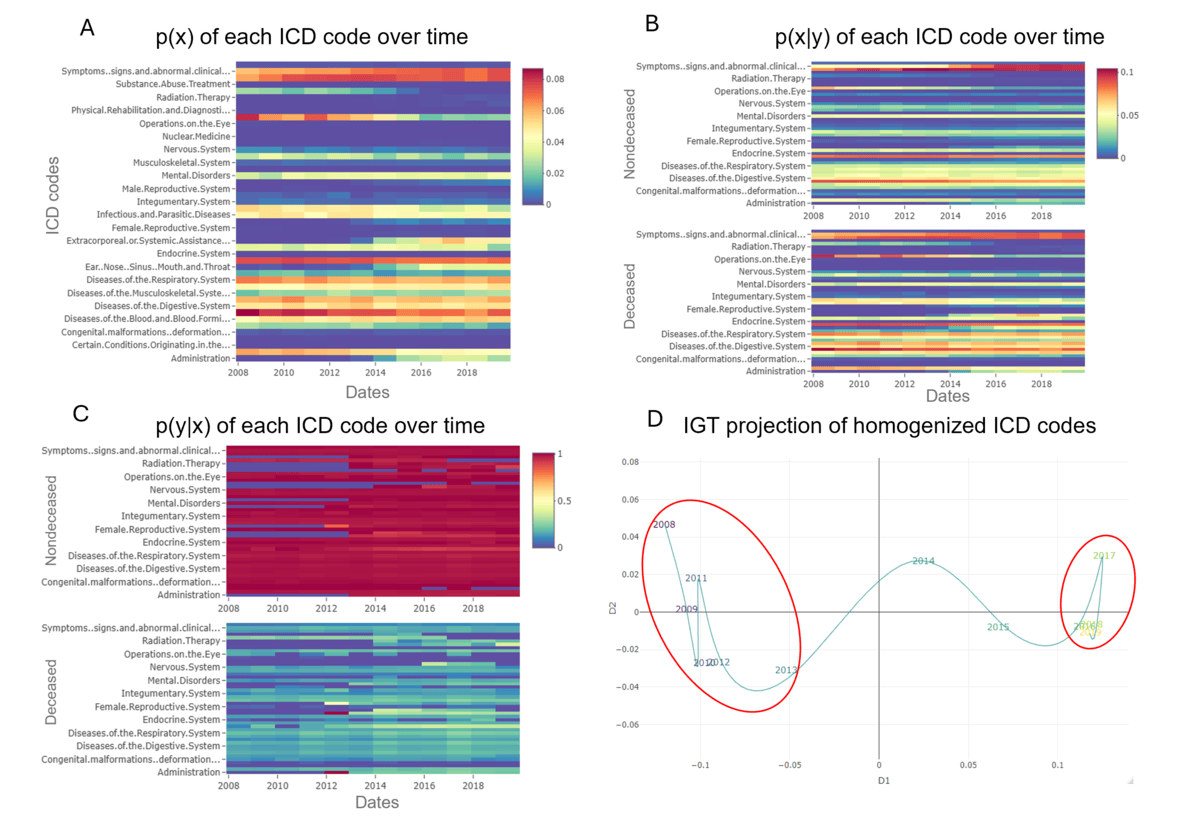
Results of the dataset shift characterization of each International Classification of Diseases (*ICD*) code, homogenized between *ICD-9* (*International Classification of Diseases, Ninth Revision*) and *ICD-10* (*International Statistical Classification of Diseases, Tenth Revision*) versions (Tables S1-S7 in Multimedia Appendix 1). (A) Heatmap of the probability distribution p(x) of each homogenized ICD code over time, sorted alphabetically. (B) Heatmap of the conditional probability distribution p(x|y) of each class for each homogenized ICD code sorted alphabetically. (C) Heatmap of the posterior probability distribution p(y|x) for each class of homogenized ICD codes, sorted alphabetically, with a square root transformation for improved visualization. (D) Information Geometric Temporal (IGT) projection of the p(x) of ICD codes. Temporal subgroups are highlighted by red circles.

Figure 3 shows the results of the univariate analysis regarding the homogenized *ICD* codes. Figure 3A shows each chapter’s overall prevalence from 2008 to 2019. Notably, “Other procedures” code diminishes its distribution over time, as it is much more present between 2008 and 2015 than in the following years. It happened similarly in the “Cardiovascular system” code. These are 2 of the most frequent chapters, both exceeding 70,000 entries. This means that variations in their distributions have a high relevance in the total variability of the dataset. The diagnosis chapters “Diseases of the circulatory system” and “Endocrine nutritional and metabolic diseases and immunity disorders” also show a diminution in the probability distribution over time. As before, these are very frequent chapters. The common fact between these chapters is that their probability distribution is higher when only *ICD-9* codes were used. This means that some of the procedures and diagnosis codes related to these chapters have been redistributed into others with the implementation of the *ICD-10*. This may have happened with the diagnoses chapter “Symptoms signs and abnormal clinical and laboratory findings not elsewhere classified” and the procedures chapters “Imaging” and “Extracorporeal or systemic assistance and performance administration,” which increased their probability distribution over time. Again, these variations are clear evidence of a covariate shift. Both the DTHs for p(x|y) and p(y|x), shown in Figures 3B and 3C, reveal synchronous changes in key chapters, such as “Administration” or “Extracorporeal or systemic assistance.” These parallel shifts in feature prevalence and their link to mortality confirm a genuine concept shift, where the relationship between certain codes and the outcome evolves over time. Figure 3D shows the IGT projection of the features’ marginal distribution, yielding 2 clear temporal subgroups (2008-2013 and 2015-2019), with a transition year.

Finally, Figure S2 in Multimedia Appendix 1 shows a stable probability for the mortality class over time, indicating no prior probability shift.

### Assessment of Models’ Performance Over Time

**Figure 4:**
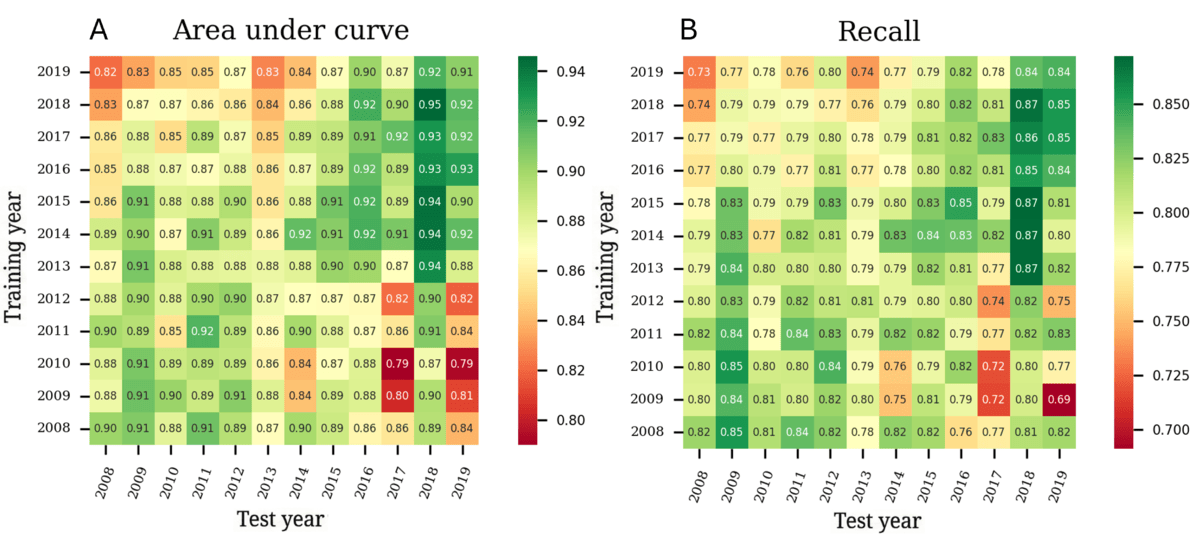
Interyear evaluation of the random forest (RF) machine learning models using the test datasets for (A) area under the receiver operating characteristic curve and (B) recall of the positive class metrics. The selected hyperparameters for the RF model were 500 trees and a maximum depth of 9.

After applying grid search for the hyperparameter tuning, the number of trees selected for the random forest models was 500 with a maximum depth of 9. In the case of gradient boosting models, the number of estimators was 100 with a maximum depth of 2. Figure 4 shows the matrices of resulting metrics from training, representing how the trained models perform when validated with data from different years. This provides insight into their behavior across temporal variations for random forest. Almost every metric described the same trend. The best results were in the lower left and upper right quadrants. These areas belonged to the models trained and tested with data batches preinstauration of *ICD-10* codes and post instauration, respectively. The remaining metrics for random forest are in Figure S3 in Multimedia Appendix 1.

As with the random forest, for gradient boosting models, every metric except precision showed better results when the models were trained and tested with pre–*ICD-10* and post–*ICD-10* data (Figure S4 in Multimedia Appendix 1). The precision metric showed very similar results in every temporal batch. Both techniques performed similarly, with slightly better results in the random forest.

### Relationship Between Temporal Variability and ML Models’ Performance

**Figure 5:**
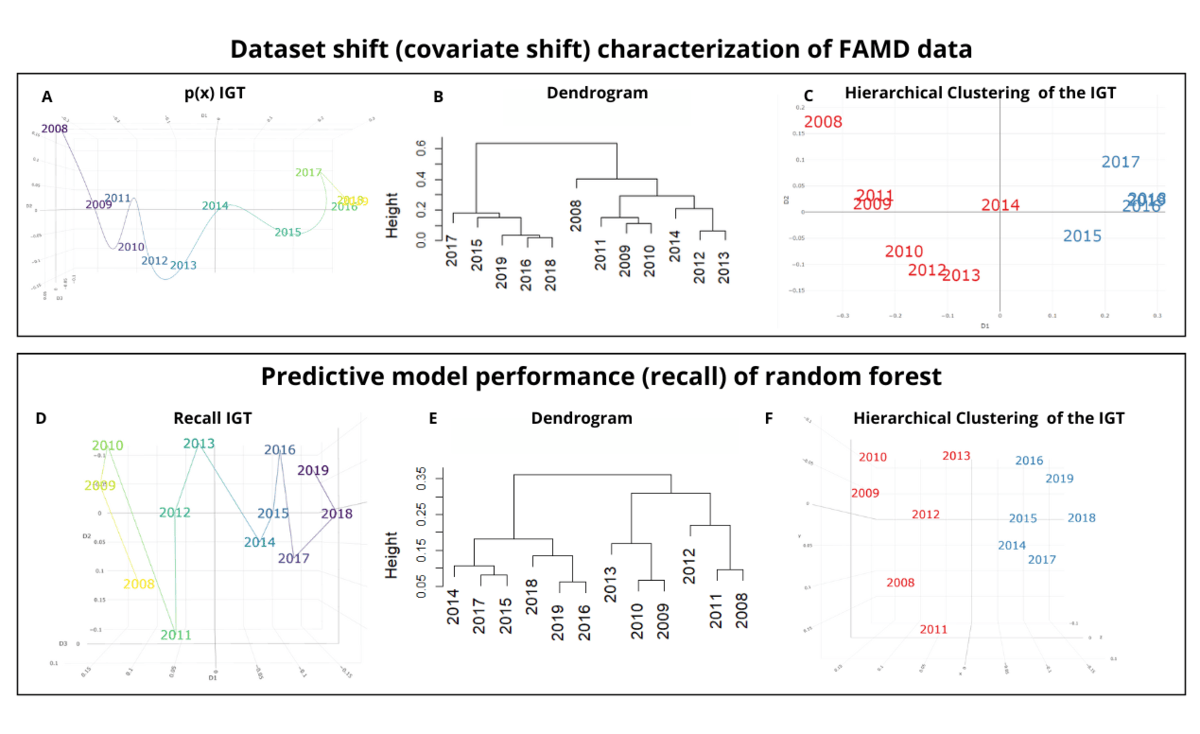
Exploratory results of comparing dataset shift characterization of factor analysis of mixed data (FAMD), data covariate shift, and random forest model evaluation matrices of recall. (A) Information Geometric Temporal (IGT) of the initial 3 dimensions of the FAMD after applying a kernel density estimation. (B) Dendrogram depicting the hierarchical clustering of the IGT for the initial 3 dimensions derived from the FAMD, following the application of kernel density estimation. (C) Representation of the hierarchical clustering of the IGT from the FAMD following the application of kernel density estimation. (D) IGT of the evaluation matrix for the recall metric of the random forest model. (E) Dendrogram depicting the hierarchical clustering of the IGT for the evaluation matrix for the recall metric on the random forest model. (F) Representation of the hierarchical clustering of the IGT from the evaluation matrix for the recall metric on the random forest model.

Figure 5 presents the exploratory results of hierarchical clustering applied to the IGT of both the dataset shift (covariate shift) characterization of the FAMD data and the recall metric from the random forest models. The remaining metrics are detailed in Figures S5-S15 in Multimedia Appendix 1. For the covariate shift (Figures 5B and 5C), the clustering results reveal 2 distinct groups (one covering 2008-2014 and the other 2015-2019), indicating similar probability distributions within these periods. These clusters suggest periods with consistent covariate characteristics, while also highlighting the presence of shifts between them. Figure 5D shows the IGT for the recall metric over time, reflecting fluctuations in model performance. The clustering results (Figures 5E and 5F) reveal 2 distinct clusters as well, with one corresponding to 2008-2013 and the other to 2014-2019. These findings illustrate how both dataset shifts and model performance evolve over time, identifying similar temporal patterns in both the data and the model behavior.

Table 1 shows the results of the Fisher exact tests comparing groups formed by hierarchical and k-means clustering of temporal variability against the metrics of random forest models for 2 clusters. All metrics registered *P* values of <.05 for both clustering methods, indicating statistically significant differences between the clusters. Specifically, these *P* values suggest that the likelihood of observing such differences by chance is less than 5%, allowing us to confidently reject the null hypothesis of independence between clusters and model performance metrics.

Similarly, Table 2 presents the results of the Fisher exact tests comparing groups formed by hierarchical and k-means clustering of temporal variability with the performance metrics of gradient boosting models across 2 clusters. In the hierarchical clustering scenario, all performance metrics except for recall exhibited *P* values <.05, indicating statistically significant differences between the clusters. This allows us to reject the null hypothesis of independence, suggesting a significant association between temporal variability and the performance metrics of gradient boosting models. For the k-means clustering, every metric recorded a *P* value of <.05. To minimize the type 1 error rate after conducting multiple statistical tests and to ensure the robustness of our results, we adjusted the *P* values using the false discovery rate method (Tables S8 and S9 in Multimedia Appendix 1). The adjusted *P* values yielded the same statistically significant results. Furthermore, the findings from the gradient boosting analysis reinforce the association between temporal variability and model performance, which aligns with the patterns observed in the random forest results. The significant *P* values for key metrics in both hierarchical and k-means clustering reinforce the conclusion that temporal variability is an important factor influencing AI model performance outcomes. These results highlight the necessity of monitoring temporal shifts in datasets to maintain and enhance the reliability and accuracy of AI models in clinical settings.

**Table 1:** Results of the Fisher exact test to compare 2 clustering methods for the random forest models (hierarchical clustering and k-means), both for *k*=2. Clustering was applied to the Information Geometric Temporal (IGT) obtained from the metrics of the random forest model and the IGT from the 3 principal dimensions of the factorial analysis of mixed data after applying the kernel density estimation. This comparison aimed to test the hypothesis of independence of both clusters (α=.05). All *P* values of <.05 are statistically significant.

Metric	*P* value for hierarchical clustering	*P* value for k-means
ROC-AUC^a^	.03	.03
PR-AUC^b^	.02	.03
Precision	.02	.03
Recall	.02	.02
Accuracy	.02	.02
*F*_1_-score	.03	.03

^a^ROC-AUC: receiver operating characteristic area under the curve.

^b^PR-AUC: precision recall area under the curve.

**Table 2:** Results of the Fisher exact test to compare 2 clustering methods for the gradient boosting model (hierarchical clustering and k-means), both for *k*=2. Clustering was applied to the Information Geometric Temporal (IGT) obtained from the metrics of the gradient boosting model and the IGT from the 3 principal dimensions of the factorial analysis of mixed data after applying the kernel density estimation. This comparison aimed to test the hypothesis of independence of both clusters (α=.05). All *P* values of <.05 are statistically significant.

Metric	*P* value for hierarchical clustering	*P* value for k-means
ROC-AUC^a^	.03	.03
PR-AUC^b^	.03	.03
Precision	.03	.02
Recall	.08	.02
Accuracy	.02	.02
*F*_1_-score	.02	.02

^a^ROC-AUC: receiver operating characteristic area under the curve.

^b^PR-AUC: precision recall area under the curve.

## Discussion

### Overview

Our study reveals a strong link between temporal dataset shifts in the MIMIC-IV dataset and the observed variations in ML performance. By using an unsupervised exploratory method to detect these shifts before model training, we can offer actionable insights regarding data curation or learning strategies (eg, domain adaptation or continual learning techniques). This early characterization not only enhances the robustness and reliability of AI models but also sets the stage for future work on online dataset shift detection. Such real-time monitoring could dynamically adjust training processes, ensuring optimal performance without frequent retraining.

When considering approaches for detecting dataset shift, there are important trade-offs between unsupervised and supervised methods. Unsupervised methods, like the one used in our study, since they are based only on data, do not require any model training nor, in the case of covariate shift analyses, labeled data. This makes them scalable and relatively cost-effective for large and dynamic clinical datasets. In contrast, supervised methods such as progressive error tracking or evaluation on external holdout sets provide direct insights into performance changes but require training particular models and computational resources. This may limit their feasibility for continuous or real-time monitoring when no labeled data are still available, as well as the inherent limitations of the selected model. Ultimately, combining both approaches may offer the most comprehensive strategy for maintaining robust AI performance in the face of evolving health data.

Our analysis of the MIMIC-IV dataset provides crucial information for understanding its characteristics and potential applications in AI. We have observed both gradual and abrupt changes in the dataset’s probability distribution over time, primarily due to variations in *ICD* codes. The transition from *ICD-9* to *ICD-10* has been the main factor contributing to this temporal variability. Even after standardizing the values, we found that this transition can significantly impact data analysis. Although mapping *ICD-9* codes to *ICD-10* codes helps reduce dimensionality and facilitates longitudinal analysis, it may not fully account for changes in coding practices or semantic shifts between the 2 versions. This issue is particularly important for chapters that have undergone significant restructuring. It has opened up new avenues for investigation, such as a thorough fidelity assessment for all chapters. Although this assessment is currently beyond the scope of this work, it may lay the groundwork for future research.

We have also discovered that the optimal number of clusters aligns with the number of *ICD* code versions. The data years are typically grouped based on the *ICD* versions used during the clustering process. Although the transition year from *ICD-9* to *ICD-10* was 2015, this study’s cutoff point was 2014. This is because the data deidentification process is not entirely precise, which is the biggest limitation in this paper, and the obtained year falls within a 3-year group. Therefore, data from 2015 may be labeled as from 2014. For this reason, we have established 2014 as the cutoff point for our association analysis (more information about the deidentification process in Section 1.1 in Multimedia Appendix 1).

It is important to note that small differences between years may arise from variations in available data, deidentification procedures, and the approximate date assignment. By following the annual extraction approach of Yao et al [9], we achieve better temporal granularity, but we must recognize that the assigned admission year may be offset by up to 1 year due to the 3-year cohort structure. As a result, the timing of observed transitions, such as the shift to *ICD-10*, should be interpreted as occurring within a 3-year window rather than as a precise annual event. This limitation may artificially sharpen the observed temporal break and exaggerate the separation between clusters in our analysis. Therefore, some of the distinct clustering observed around the *ICD* system transition could reflect this methodological artifact rather than a purely organic change in the underlying data distribution. To assess the impact of this limitation, we repeated our analysis using the original 3-year cohort groups instead of annual assignments and observed that our main conclusions regarding dataset shifts and their effects on model performance remained unchanged. This suggests that our findings are robust to the temporal aggregation method.

Importantly, we showed that both linear (FAMD) and nonlinear (UMAP) dimensionality reduction techniques produce consistent temporal clusters, supporting the robustness of our findings despite FAMD capturing only 15.16% of total variance. This robustness underscores the value of unsupervised characterization for understanding temporal variability. It suggests that, despite the relatively low explained variance of the FAMD approach, our main conclusions regarding temporal dataset shifts remain stable across both linear and nonlinear dimensionality reduction techniques.

Recent studies have further emphasized the importance of considering temporal shifts in health AI. Peracchio et al [35] and Nicora et al [36] focused on performing an online detection of temporal changes in clinical settings during the deployment phase of AI models, highlighting how these shifts impact AI model performance, which aligns with our findings. Saurav et al [37] contributed to this field by performing an anomaly detection methodology using recurrent neural networks to make multistep time series predictions and using the prediction errors to detect shifts and rapidly adapt the model to changes. Furthermore, Koch et al [38] have shown that detecting dataset shifts is crucial for monitoring AI-based medical products during postmarket surveillance, implementing 3 different methods to do it. While Guo et al [39] also used an unsupervised approach to investigate dataset shifts in the MIMIC-IV dataset, our study differs by focusing on a model-agnostic and exploratory characterization of these shifts. Our approach is computationally simpler and offers enhanced flexibility and scalability. Additionally, our study explores how unsupervised methods can interact with supervised approaches, offering complementary insights into the identification of temporal variability.

Overall, our work builds upon existing literature on the impact of temporal variability and dataset shifts on ML models. By adopting an unsupervised, model-agnostic approach based on information geometry, we contribute to a growing body of research seeking to address the challenges posed by temporal shifts in clinical AI. Our findings enhance the understanding of how these shifts influence model robustness and performance.

This approach not only optimizes decision-making in clinical practice but also enhances the adaptability of AI systems. By enabling early characterization of dataset shifts, our work can guide clinicians and developers in identifying emerging trends and anticipating potential risks. Ultimately, this work lays the groundwork for the development of continuously learning, self-adaptive AI models that are better equipped to handle the dynamic nature of health data.

### Conclusions

This work demonstrates that early identification of changes in data statistical distributions through unsupervised dataset shift characterization is a potential indicator of performance variations in prospective ML models. The association between variations in data distributions and the model’s performance has been statistically demonstrated in the MIMIC-IV dataset as a significant benchmark. Noteworthy, the implementation of *ICD-10* coding resulted in significant temporal variability in covariates and outcome-conditional distributions. This translates into the presence of covariate and concept shift, but not prior probability shift in the dataset.

Our findings underscore the importance of characterizing and adjusting for dataset shifts when developing AI models in health. By integrating temporal variability analysis, such as IGT projection data, with the development of AI models, we pave the way to the creation of trustworthy and self-adaptive AI systems. Our research sets the stage for building more resilient and adaptable AI solutions, which will help enhance patient safety and increase the applicability of AI in the ever-evolving health industry.

## Appendix

Multimedia Appendix 1 Additional information, tables, and figures.

## Abbreviations

AI: artificial intelligence
BIDMC: Beth Israel Deaconess Medical Center
DTH: data temporal heatmaps
EHR: electronic health record
FAMD: factorial analysis of mixed data
ICD: International Classification of Diseases
ICD-9: International Classification of Diseases, Ninth Revision
ICD-10: International Statistical Classification of Diseases, Tenth Revision
IGT: Information Geometric Temporal
KDE: kernel density estimation
MIMIC-IV: Medical Information Mart for Intensive Care-IV
ML: machine learning
ROC-AUC: receiver operating characteristic area under the curve
UMAP: Uniform Manifold Approximation and Projection
