# Supplementary material for: Unsupervised Characterization of Temporal Dataset Shifts as an Early Indicator of AI Performance Variations: Evaluation Study Using the Medical Information Mart for Intensive Care-IV Dataset
Source: JMIR Med Inform. 2025 Dec 3;13:e78309. doi: 10.2196/78309 (PMC12712564; doi:10.2196/78309)
Supplement: Multimedia Appendix 1 [file medinform_v13i1e78309_app1.docx]

**SUPPLEMENTARY INFORMATION**

**1.1**

The MIMIC-IV dataset encodes temporal information in two variables to ensure data anonymity. The first variable, anchor_year, is a deidentified year ranging between 210 0 and 2200. The second variable, anchor_year_group, categorizes admissions into 3-year cohorts spanning from 2008 to 2019 (e.g., 2008–2010, 2011–2013, 2014–2016, etc.). This grouping facilitates the approximation of the actual admission year for each patient while maintaining anonymity. Additionally, the admittime variable mirrors the structure of anchor_year, providing a timestamp for each admission. To achieve yearly temporal granularity for our analysis, we applied a preprocessing method adapted from Yao et al. (Wild-Time: A Benchmark of in-the-Wild Distribution Shift over Time, NeurIPS 2022). This preprocessing involves calculating the approximate_actual_date using the following formula:

| $approximate\_actual\_date=admittime-anchor\_year +real\_anchor\_year$ | (1) |
| --- | --- |

Here, real_anchor_year represents the first year of the corresponding 3-year cohort defined by anchor_year_group. For instance, admissions within the 2014–2016 cohort use 2014 as the real_anchor_year. As a result, all admissions in a given cohort are assigned the same anchor year (the first in the group), even though the true admission could have occurred in any of the three years. Thus, the assigned year may be up to ±1 year from the true date. It is important to note that the designation of 2014 as the ICD-10 transition year was a result of the 3-year cohort grouping, rather than an intentional selection. Due to this grouping, data from both 2015 and 2016 may also be labeled as 2014. This temporal imprecision means that the true timing of observed distribution shifts—such as the ICD-10 transition—could occur anywhere within the 2014–2016 window. This methodological choice was made to balance temporal resolution with data anonymization requirements.

**Table S1: Chapters of ICD-9 diagnoses**

| ICD-9 | Group |
| --- | --- |
| [001-139](https://dexur.com/icd9/001-139/) | Infectious And Parasitic Diseases |
| [140-239](https://dexur.com/icd9/140-239/) | Neoplasms |
| [240-279](https://dexur.com/icd9/240-279/) | Endocrine, Nutritional And Metabolic Diseases, And Immunity Disorders |
| [280-289](https://dexur.com/icd9/280-289/) | Diseases Of The Blood And Blood-Forming Organs |
| [290-319](https://dexur.com/icd9/290-319/) | Mental Disorders |
| [320-389](https://dexur.com/icd9/320-389/) | Diseases Of The Nervous System And Sense Organs |
| [390-459](https://dexur.com/icd9/390-459/) | Diseases Of The Circulatory System |
| [460-519](https://dexur.com/icd9/460-519/) | Diseases Of The Respiratory System |
| [520-579](https://dexur.com/icd9/520-579/) | Diseases Of The Digestive System |
| [580-629](https://dexur.com/icd9/580-629/) | Diseases Of The Genitourinary System |
| [630-679](https://dexur.com/icd9/630-679/) | Complications Of Pregnancy, Childbirth, And The Puerperium |
| [680-709](https://dexur.com/icd9/680-709/) | Diseases Of The Skin And Subcutaneous Tissue |
| [710-739](https://dexur.com/icd9/710-739/) | Diseases Of The Musculoskeletal System And Connective Tissue |
| [740-759](https://dexur.com/icd9/740-759/) | Congenital Anomalies |
| [760-779](https://dexur.com/icd9/760-779/) | Certain Conditions Originating In The Perinatal Period |
| [780-799](https://dexur.com/icd9/780-799/) | Symptoms, Signs, And Ill-Defined Conditions |
| [800-999](https://dexur.com/icd9/800-999/) | Injury And Poisoning |
| [V01-V91](https://dexur.com/icd9/V01-V91/) | Supplementary Classification Of Factors Influencing Health Status And Contact With Health Services |
| [E000-E999](https://dexur.com/icd9/E000-E999/) | Supplementary Classification Of External Causes Of Injury And Poisoning |

**Table S2: Chapters of ICD-10 diagnoses**

| ICD-10 | Group |
| --- | --- |
| [A00-B99](https://dexur.com/icd10/A00-B99/) | Certain infectious and parasitic diseases |
| [C00-D49](https://dexur.com/icd10/C00-D49/) | Neoplasms |
| [D50-D89](https://dexur.com/icd10/D50-D89/) | Diseases of the blood and blood-forming organs and certain disorders involving the immune mechanism |
| [E00-E89](https://dexur.com/icd10/E00-E89/) | Endocrine, nutritional and metabolic diseases |
| [F01-F99](https://dexur.com/icd10/F01-F99/) | Mental, Behavioral and Neurodevelopmental disorders |
| [G00-G99](https://dexur.com/icd10/G00-G99/) | Diseases of the nervous system |
| [H00-H59](https://dexur.com/icd10/H00-H59/) | Diseases of the eye and adnexa |
| [H60-H95](https://dexur.com/icd10/H60-H95/) | Diseases of the ear and mastoid process |
| [I00-I99](https://dexur.com/icd10/I00-I99/) | Diseases of the circulatory system |
| [J00-J99](https://dexur.com/icd10/J00-J99/) | Diseases of the respiratory system |
| [K00-K95](https://dexur.com/icd10/K00-K95/) | Diseases of the digestive system |
| [L00-L99](https://dexur.com/icd10/L00-L99/) | Diseases of the skin and subcutaneous tissue |
| [M00-M99](https://dexur.com/icd10/M00-M99/) | Diseases of the musculoskeletal system and connective tissue |
| [N00-N99](https://dexur.com/icd10/N00-N99/) | Diseases of the genitourinary system |
| [O00-O9A](https://dexur.com/icd10/O00-O9A/) | Pregnancy, childbirth and the puerperium |
| [P00-P96](https://dexur.com/icd10/P00-P96/) | Certain conditions originating in the perinatal period |
| [Q00-Q99](https://dexur.com/icd10/Q00-Q99/) | Congenital malformations, deformations and chromosomal abnormalities |
| [R00-R99](https://dexur.com/icd10/R00-R99/) | Symptoms, signs and abnormal clinical and laboratory findings, not elsewhere classified |
| [S00-T88](https://dexur.com/icd10/S00-T88/) | Injury, poisoning and certain other consequences of external causes |
| [V00-Y99](https://dexur.com/icd10/V00-Y99/) | External causes of morbidity |
| [Z00-Z99](https://dexur.com/icd10/Z00-Z99/) | Factors influencing health status and contact with health services |

**Table S3: Homogenized diagnoses chapters**

| Grouping ICD-9 ICD-10 | Correspondence ICD-9 | Correspondence ICD-10 |
| --- | --- | --- |
| Infectious and Parasitic Diseases | [001-139](https://dexur.com/icd9/001-139/) | [A00-B99](https://dexur.com/icd10/A00-B99/) |
| Neoplasms | [140-239](https://dexur.com/icd9/140-239/) | [C00-D49](https://dexur.com/icd10/C00-D49/) |
| Endocrine, Nutritional and Metabolic Diseases, and Immunity Disorders | [240-279](https://dexur.com/icd9/240-279/) | [E00-E89](https://dexur.com/icd10/E00-E89/) |
| Diseases of the Blood and Blood-Forming Organs | [280-289](https://dexur.com/icd9/280-289/) | [D50-D89](https://dexur.com/icd10/D50-D89/) |
| Mental Disorders | [290-319](https://dexur.com/icd9/290-319/) | [F01-F99](https://dexur.com/icd10/F01-F99/) |
| Diseases of the Nervous System and Sense Organs | [320-389](https://dexur.com/icd9/320-389/) | [G00-G99, H00-H59, H60-H95](https://dexur.com/icd10/G00-G99/) |
| Diseases of the Circulatory System | [390-459](https://dexur.com/icd9/390-459/) | [I00-I99](https://dexur.com/icd10/I00-I99/) |
| Diseases of the Respiratory System | [460-519](https://dexur.com/icd9/460-519/) | [J00-J99](https://dexur.com/icd10/J00-J99/) |
| Diseases of the Digestive System | [520-579](https://dexur.com/icd9/520-579/) | [K00-K95](https://dexur.com/icd10/K00-K95/) |
| Diseases of the Genitourinary System | [580-629](https://dexur.com/icd9/580-629/) | [N00-N99](https://dexur.com/icd10/N00-N99/) |
| Complications of Pregnancy, Childbirth, and the Puerperium | [630-679](https://dexur.com/icd9/630-679/) | [O00-O9A](https://dexur.com/icd10/O00-O9A/) |
| Diseases of the Skin and Subcutaneous Tissue | [680-709](https://dexur.com/icd9/680-709/) | [L00-L99](https://dexur.com/icd10/L00-L99/) |
| Diseases of the Musculoskeletal System and Connective Tissue | [710-739](https://dexur.com/icd9/710-739/) | [M00-M99](https://dexur.com/icd10/M00-M99/) |
| Congenital malformations, deformations and chromosomal abnormalities | [740-759](https://dexur.com/icd9/740-759/) | [Q00-Q99](https://dexur.com/icd10/Q00-Q99/) |
| Certain Conditions Originating in the Perinatal Period | [760-779](https://dexur.com/icd9/760-779/) | [P00-P96](https://dexur.com/icd10/P00-P96/) |
| Symptoms, signs and abnormal clinical and laboratory findings, not elsewhere classified | [780-799](https://dexur.com/icd9/780-799/) | [R00-R99](https://dexur.com/icd10/R00-R99/) |
| Injury and Poisoning | [800-999](https://dexur.com/icd9/800-999/) | [S00-T88](https://dexur.com/icd10/S00-T88/) |
| Supplementary Classification of Factors Influencing Health Status and Contact with Health Services | [V01-V91](https://dexur.com/icd9/V01-V91/) | [Z00-Z99](https://dexur.com/icd10/Z00-Z99/) |
| External causes of morbidity | [E000-E999](https://dexur.com/icd9/E000-E999/) | [V00-Y99](https://dexur.com/icd10/V00-Y99/) |

**Table S4: Chapters of ICD-9 procedures**

| ICD-9 | Group |
| --- | --- |
| [00-00](https://dexur.com/pcs9/00-00/) | Procedures And Interventions , Not Elsewhere Classified |
| [01-05](https://dexur.com/pcs9/01-05/) | Operations On The Nervous System |
| [06-07](https://dexur.com/pcs9/06-07/) | Operations On The Endocrine System |
| [08-16](https://dexur.com/pcs9/08-16/) | Operations On The Eye |
| [17-17](https://dexur.com/pcs9/17-17/) | Other Miscellaneous Diagnostic And Therapeutic Procedures |
| [18-20](https://dexur.com/pcs9/18-20/) | Operations On The Ear |
| [21-29](https://dexur.com/pcs9/21-29/) | Operations On The Nose, Mouth, And Pharynx |
| [30-34](https://dexur.com/pcs9/30-34/) | Operations On The Respiratory System |
| [35-39](https://dexur.com/pcs9/35-39/) | Operations On The Cardiovascular System |
| [40-41](https://dexur.com/pcs9/40-41/) | Operations On The Hemic And Lymphatic System |
| [42-54](https://dexur.com/pcs9/42-54/) | Operations On The Digestive System |
| [55-59](https://dexur.com/pcs9/55-59/) | Operations On The Urinary System |
| [60-64](https://dexur.com/pcs9/60-64/) | Operations On The Male Genital Organs |
| [65-71](https://dexur.com/pcs9/65-71/) | Operations On The Female Genital Organs |
| [72-75](https://dexur.com/pcs9/72-75/) | Obstetrical Procedures |
| [76-84](https://dexur.com/pcs9/76-84/) | Operations On The Musculoskeletal System |
| [85-86](https://dexur.com/pcs9/85-86/) | Operations On The Integumentary System |
| [87-99](https://dexur.com/pcs9/87-99/) | Miscellaneous Diagnostic And Therapeutic Procedures |

**Table S5: Chapters of ICD-10 procedures**

| ICD-10 | Group |
| --- | --- |
| [0](https://dexur.com/pcs10/0/) | Medical and Surgical |
| [1](https://dexur.com/pcs10/1/) | Obstetrics |
| [2](https://dexur.com/pcs10/2/) | Placement |
| [3](https://dexur.com/pcs10/3/) | Administration |
| [4](https://dexur.com/pcs10/4/) | Measurement and Monitoring |
| [5](https://dexur.com/pcs10/5/) | Extracorporeal or Systemic Assistance and Performance |
| [6](https://dexur.com/pcs10/6/) | Extracorporeal or Systemic Therapies |
| [7](https://dexur.com/pcs10/7/) | Osteopathic |
| [8](https://dexur.com/pcs10/8/) | Other Procedures |
| [9](https://dexur.com/pcs10/9/) | Chiropractic |
| [B](https://dexur.com/pcs10/B/) | Imaging |
| [C](https://dexur.com/pcs10/C/) | Nuclear Medicine |
| [D](https://dexur.com/pcs10/D/) | Radiation Therapy |
| [F](https://dexur.com/pcs10/F/) | Physical Rehabilitation and Diagnostic Audiology |
| [G](https://dexur.com/pcs10/G/) | Mental Health |
| [H](https://dexur.com/pcs10/H/) | Substance Abuse Treatment |
| [X](https://dexur.com/pcs10/X/) | New Technology |

**Table S6: Homogenized procedures chapters without to the division of Medical and Surgical into subchapters**

| Grouping ICD-9 ICD-10 | Correspondence ICD-9 | Correspondence ICD-10 |
| --- | --- | --- |
| Medical and Surgical | [01-05, 06-07, 08-16, 18-20, 21-29, 30-34, 35-39, 40-41, 42-54, 55-59, 60-64, 65-71, 76-84, 85-86](https://dexur.com/pcs9/00-00/) | [0](https://dexur.com/pcs10/0/) |
| Obstetrical Procedures | [72-75](https://dexur.com/pcs9/72-75/) | [1](https://dexur.com/pcs10/1/) |
| Placement |  | [2](https://dexur.com/pcs10/2/) |
| Administration |  | [3](https://dexur.com/pcs10/3/) |
| Measurement and Monitoring |  | [4](https://dexur.com/pcs10/4/) |
| Extracorporeal or Systemic Assistance and Performance |  | [5](https://dexur.com/pcs10/5/) |
| Extracorporeal or Systemic Therapies |  | [6](https://dexur.com/pcs10/6/) |
| Osteopathic |  | [7](https://dexur.com/pcs10/7/) |
| Other Procedures | [17-17, 87-99, 00-00](https://dexur.com/pcs9/17-17/) | [8, 0W, 0X, 0Y](https://dexur.com/pcs10/8/) |
| Chiropractic |  | [9](https://dexur.com/pcs10/9/) |
| Imaging |  | [B](https://dexur.com/pcs10/B/) |
| Nuclear Medicine |  | [C](https://dexur.com/pcs10/C/) |
| Radiation Therapy |  | [D](https://dexur.com/pcs10/D/) |
| Physical Rehabilitation and Diagnostic Audiology |  | [F](https://dexur.com/pcs10/F/) |
| Mental Health |  | [G](https://dexur.com/pcs10/G/) |
| Substance Abuse Treatment |  | [H](https://dexur.com/pcs10/H/) |
| New Technology |  | [X](https://dexur.com/pcs10/X/) |

**Table S7: Division of homogenized Medical and Surgical chapter into subchapters**

| Medical and Surgical | Correspondence ICD-9 | Correspondence ICD-10 |
| --- | --- | --- |
| Nervous System | [01-05](https://dexur.com/pcs9/01-05/) | [00, 01](https://dexur.com/pcs10/00/) |
| Endocrine System | [06-07](https://dexur.com/pcs9/06-07/) | [0G](https://dexur.com/pcs10/0G/) |
| Operations on the Eye | [08-16](https://dexur.com/pcs9/08-16/) | [08](https://dexur.com/pcs10/08/) |
| Ear, Nose, Sinus, Mouth and Throat | [18-20, 21-29](https://dexur.com/pcs9/18-20/) | [09, 0C](https://dexur.com/pcs10/09/) |
| Respiratory System | [30-34](https://dexur.com/pcs9/30-34/) | [0B](https://dexur.com/pcs10/0B/) |
| Cardiovascular System | [35-39](https://dexur.com/pcs9/35-39/) | [02, 03, 04, 05, 06](https://dexur.com/pcs10/02/) |
| Lymphatic and Hemic Systems | [40-41](https://dexur.com/pcs9/40-41/) | [07](https://dexur.com/pcs10/07/) |
| Digestive System | [42-54](https://dexur.com/pcs9/42-54/) | [0D, 0F](https://dexur.com/pcs10/0D/) |
| Urinary System | [55-59](https://dexur.com/pcs9/55-59/) | [0T](https://dexur.com/pcs10/0T/) |
| Male Reproductive System | [60-64](https://dexur.com/pcs9/60-64/) | [0V](https://dexur.com/pcs10/0V/) |
| Female Reproductive System | [65-71](https://dexur.com/pcs9/65-71/) | [0U](https://dexur.com/pcs10/0U/) |
| Musculoskeletal System | [76-84](https://dexur.com/pcs9/76-84/) | [0K, 0L, 0M, 0N, 0P, 0Q, 0R, 0S](https://dexur.com/pcs10/0K/) |
| Integumentary System | [85-86](https://dexur.com/pcs9/85-86/) | [0H, 0J](https://dexur.com/pcs10/0H/) |


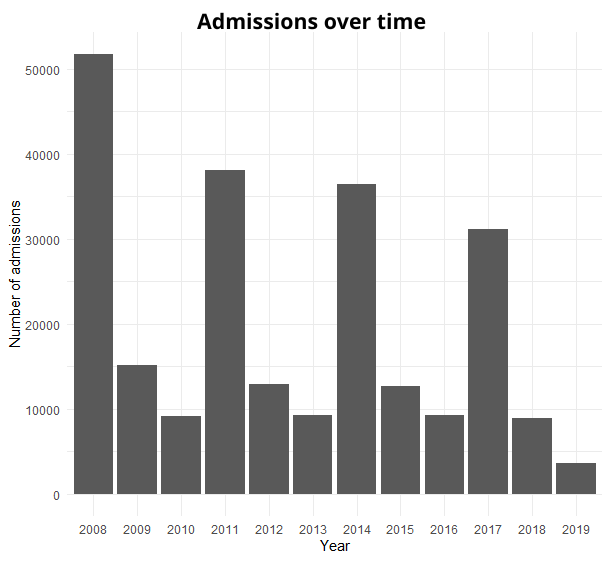


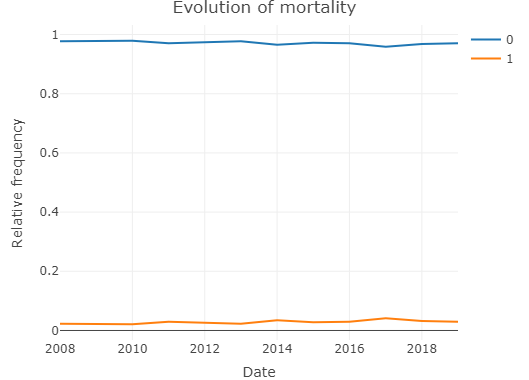


**Figure S1: Number of admissions over time in our MIMIC-IV dataset**

**Figure S2: Evolution of the mortality p(y) over time in our MIMIC-IV dataset**


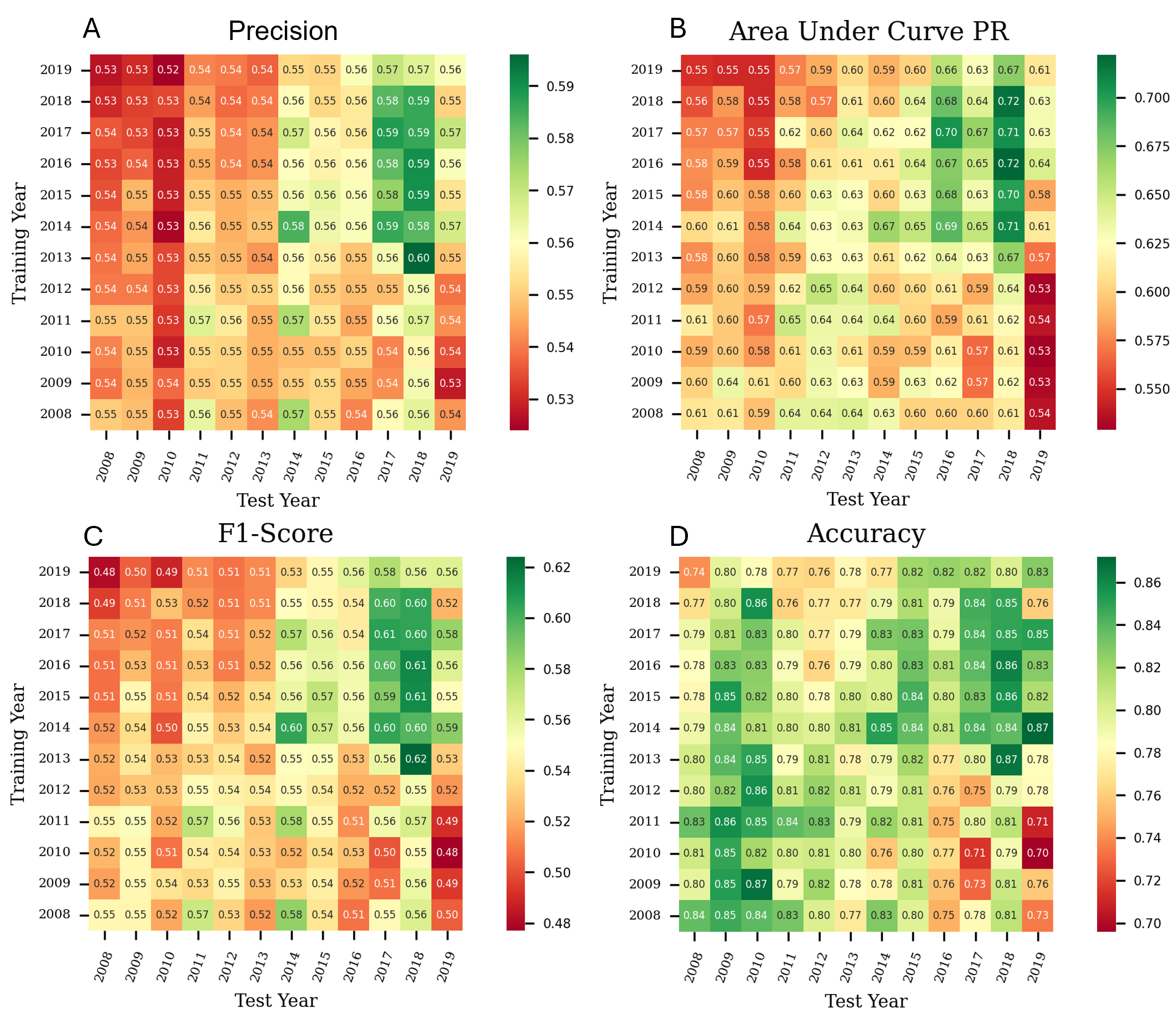


**Figure S3: Inter-year evaluation of the Random Forest (RF) machine learning models using the test data sets for Precision (A), PR-AUC (B), F1-Score (C), and Accuracy (D) metrics.** The selected hyperparameters for the RF model were 500 trees and a maximum depth of 9.


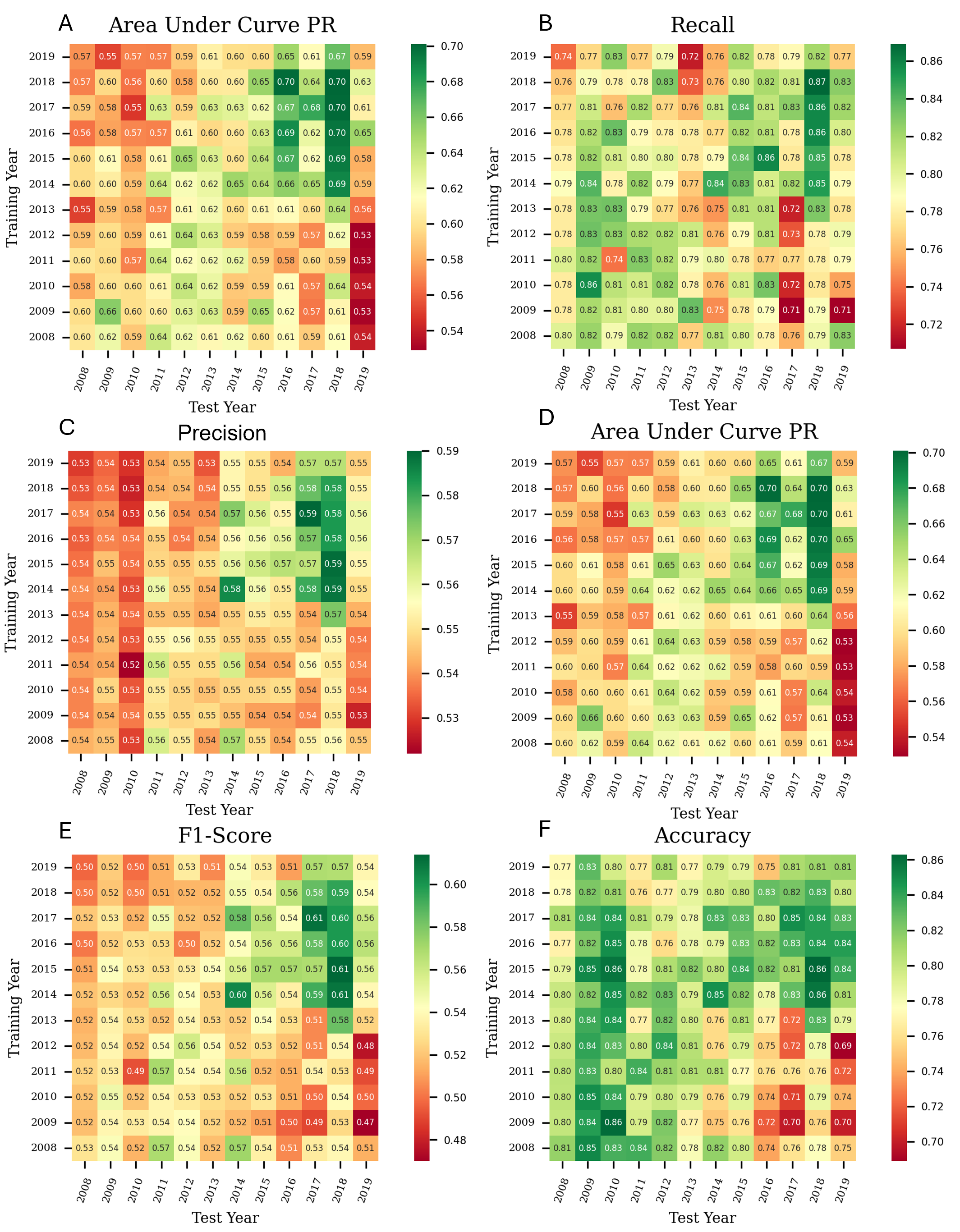


**Figure S4: Inter-year evaluation of the Gradient Boosting (GB) machine learning models using the test data sets for ROC-AUC (A), Recall (B), Precision (C), PR-AUC (D), F1-Score (E), and Accuracy (F) metrics.** The selected hyperparameters for the GB model were 100 estimators and a maximum depth of 2.


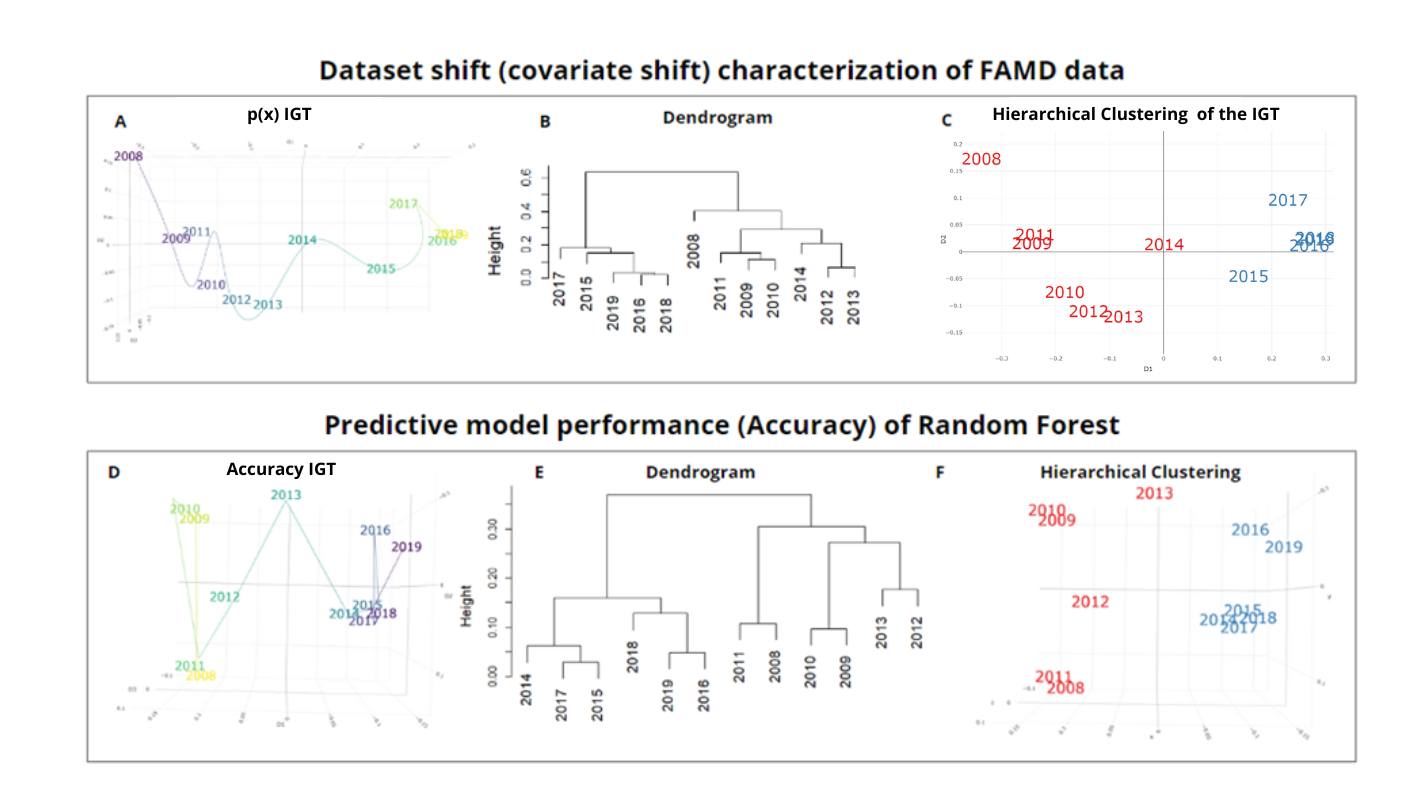

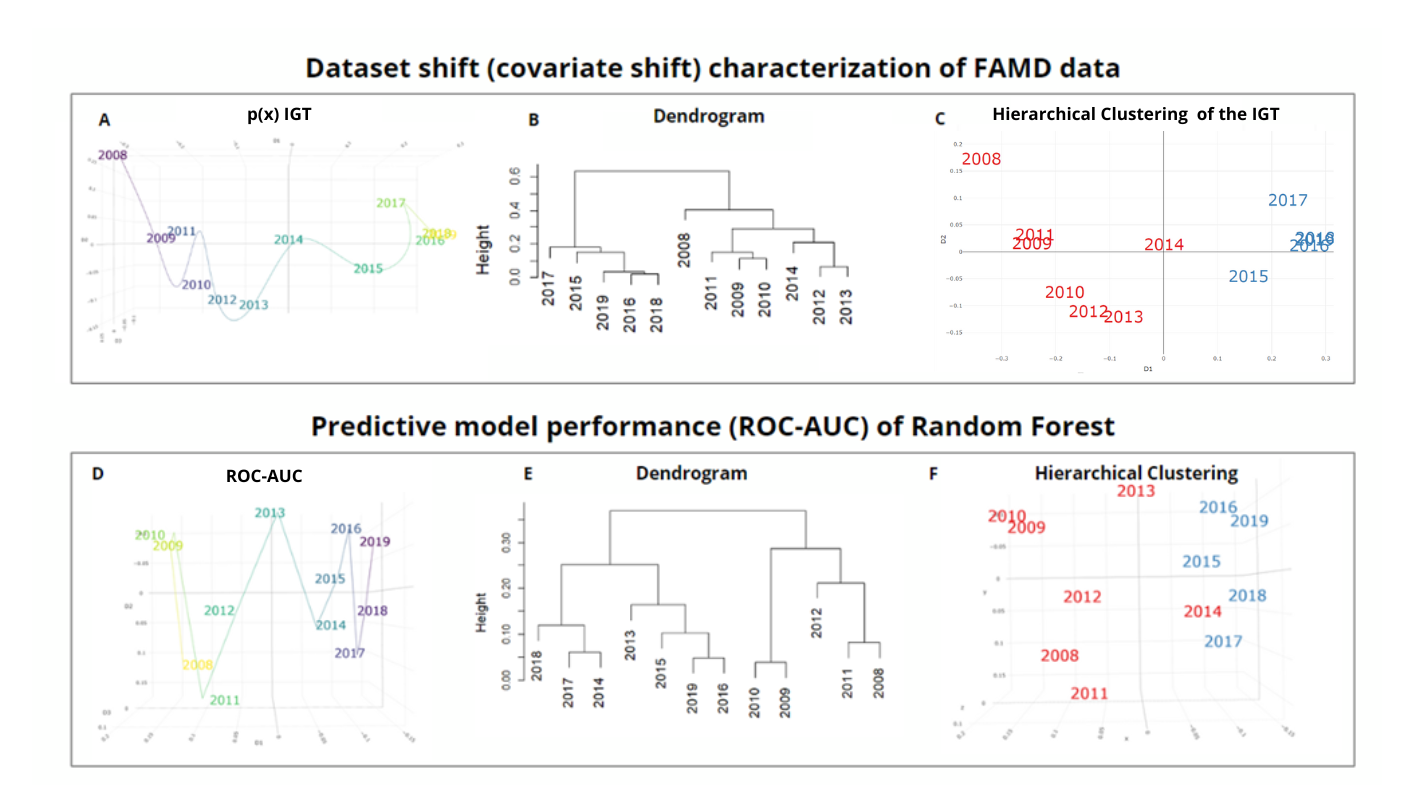


**Figure S5: Exploratory results of comparing dataset shift characterization of FAMD data covariate shift and Random Forest model evaluation matrices of ROC-AUC. A** Information Geometric Temporal (IGT) of the initial 3 dimensions of the FAMD after applying a Kernel Density Estimation. **B** Dendrogram depicting the hierarchical clustering of the IGT for the initial three dimensions derived from the Factor Analysis of Mixed Data (FAMD), following the application of Kernel Density Estimation. **C** Representation of the hierarchical clustering of the IGT from the FAMD following the application of Kernel Density Estimation. **D** IGT of the evaluation matrix for the ROC-AUC metric of the Random Forest model**.** **E** Dendrogram depicting the hierarchical clustering of the IGT for the evaluation matrix for the ROC-AUC metric on the Random Forest model. **F** Representation of the hierarchical clustering of the IGT from the evaluation matrix for the ROC-AUC metric on the Random Forest model


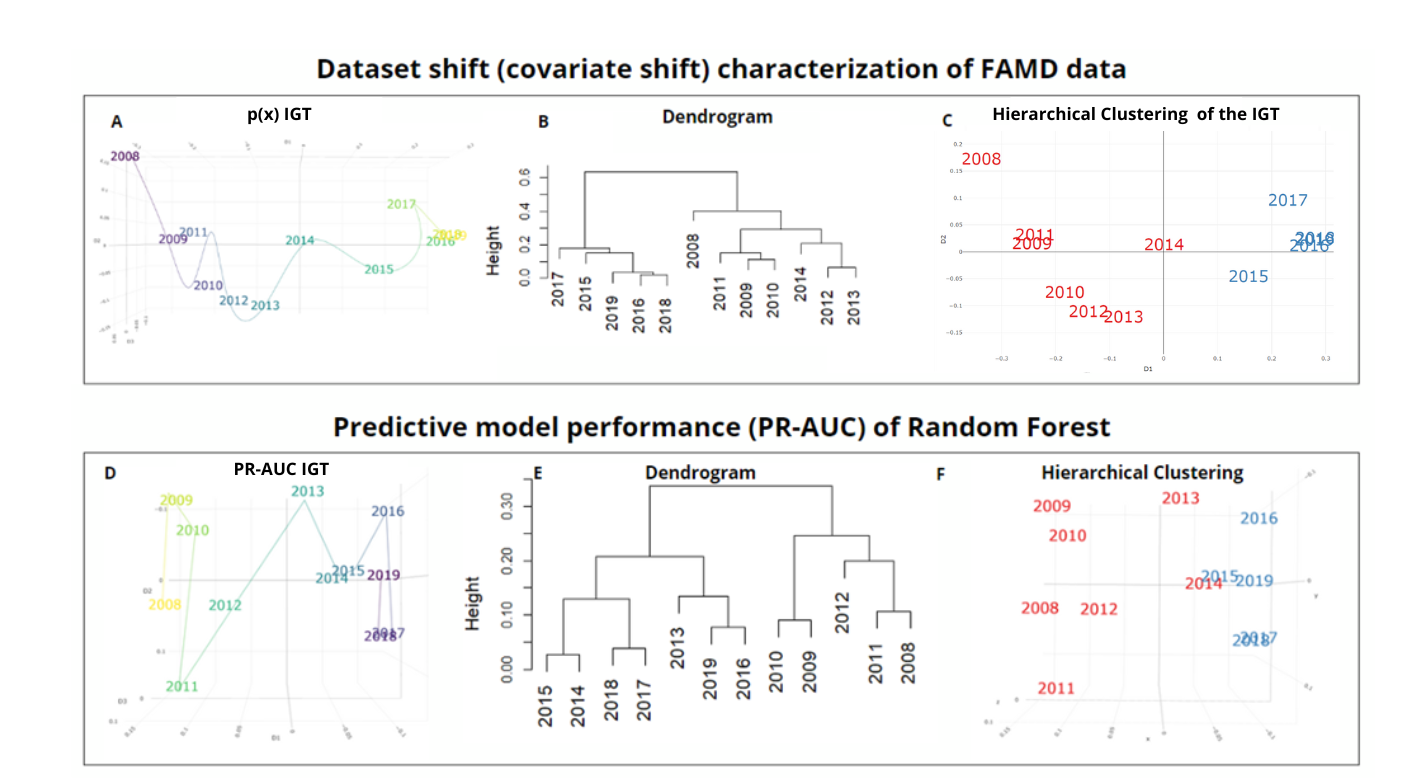


**Figure S7: Exploratory results of comparing dataset shift characterization of FAMD data covariate shift and Random Forest model evaluation matrices of PR-AUC. A** Information Geometric Temporal (IGT) of the initial 3 dimensions of the FAMD after applying a Kernel Density Estimation. **B** Dendrogram depicting the hierarchical clustering of the IGT for the initial three dimensions derived from the Factor Analysis of Mixed Data (FAMD), following the application of Kernel Density Estimation. **C** Representation of the hierarchical clustering of the IGT from the FAMD following the application of Kernel Density Estimation. **D** IGT of the evaluation matrix for the PR-AUC metric of the Random Forest model**.** **E** Dendrogram depicting the hierarchical clustering of the IGT for the evaluation matrix for the PR-AUC metric on the Random Forest model. **F** Representation of the hierarchical clustering of the IGT from the evaluation matrix for the PR-AUC metric on the Random Forest model

**Figure S6: Exploratory results of comparing dataset shift characterization of FAMD data covariate shift and Random Forest model evaluation matrices of Accuracy. A** Information Geometric Temporal (IGT) of the initial 3 dimensions of the FAMD after applying a Kernel Density Estimation. **B** Dendrogram depicting the hierarchical clustering of the IGT for the initial three dimensions derived from the Factor Analysis of Mixed Data (FAMD), following the application of Kernel Density Estimation. **C** Representation of the hierarchical clustering of the IGT from the FAMD following the application of Kernel Density Estimation. **D** IGT of the evaluation matrix for the Accuracy metric of the Random Forest model**.** **E** Dendrogram depicting the hierarchical clustering of the IGT for the evaluation matrix for the Accuracy metric on the Random Forest model. **F** Representation of the hierarchical clustering of the IGT from the evaluation matrix for the Accuracy metric on the Random Forest model


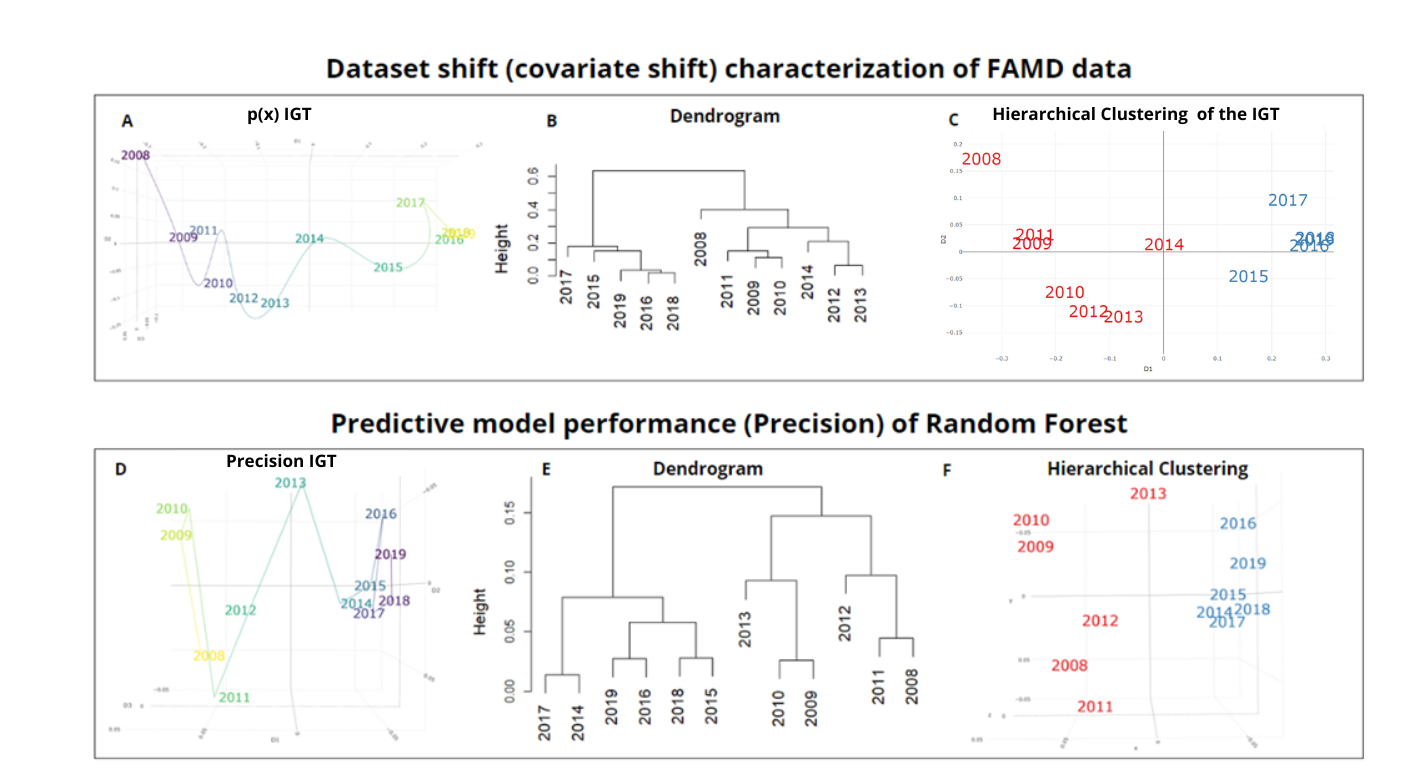

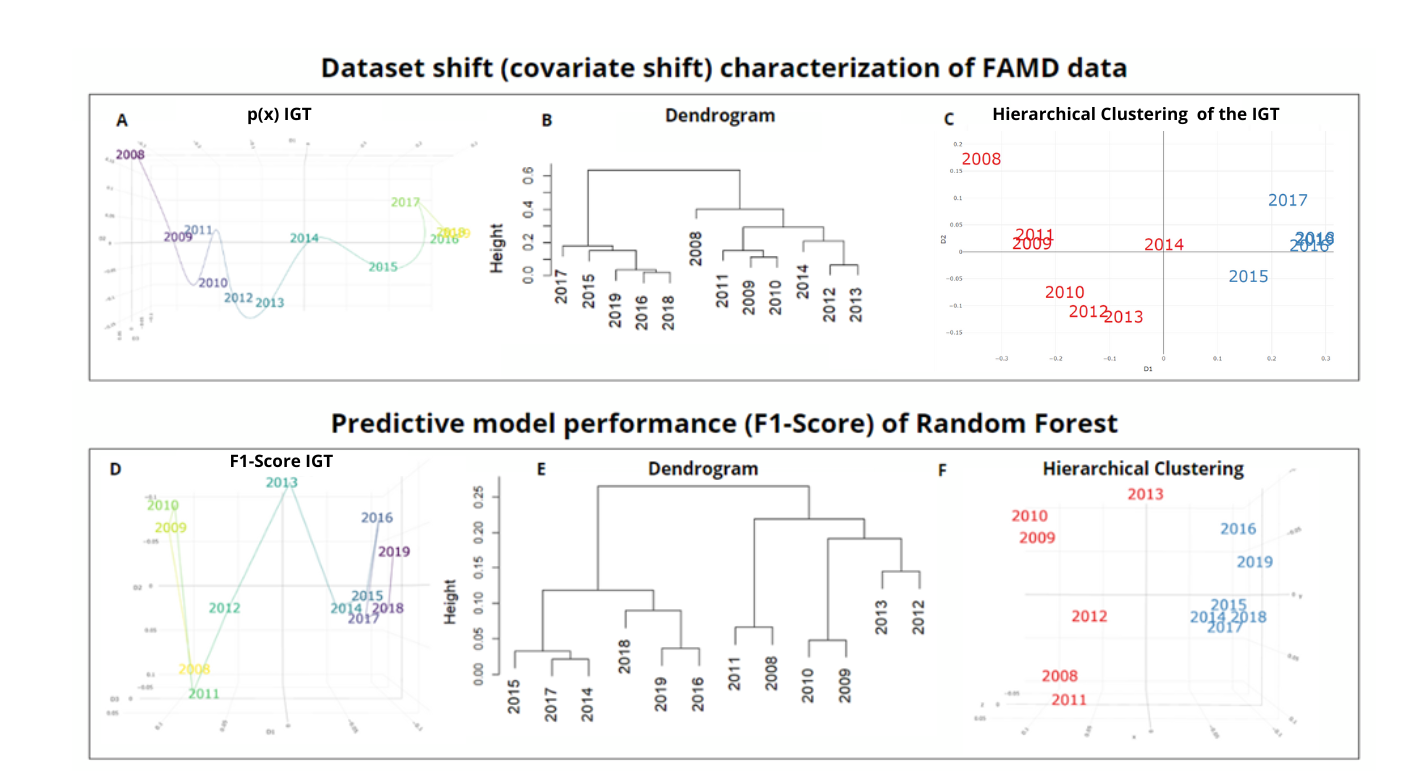


**Figure S8: Exploratory results of comparing dataset shift characterization of FAMD data covariate shift and Random Forest model evaluation matrices of F1-Score. A** Information Geometric Temporal (IGT) of the initial 3 dimensions of the FAMD after applying a Kernel Density Estimation. **B** Dendrogram depicting the hierarchical clustering of the IGT for the initial three dimensions derived from the Factor Analysis of Mixed Data (FAMD), following the application of Kernel Density Estimation. **C** Representation of the hierarchical clustering of the IGT from the FAMD following the application of Kernel Density Estimation. **D** IGT of the evaluation matrix for the F1-Score metric of the Random Forest model**.** **E** Dendrogram depicting the hierarchical clustering of the IGT for the evaluation matrix for the F1-Score metric on the Random Forest model. **F** Representation of the hierarchical clustering of the IGT from the evaluation matrix for the F1-Score metric on the Random Forest model


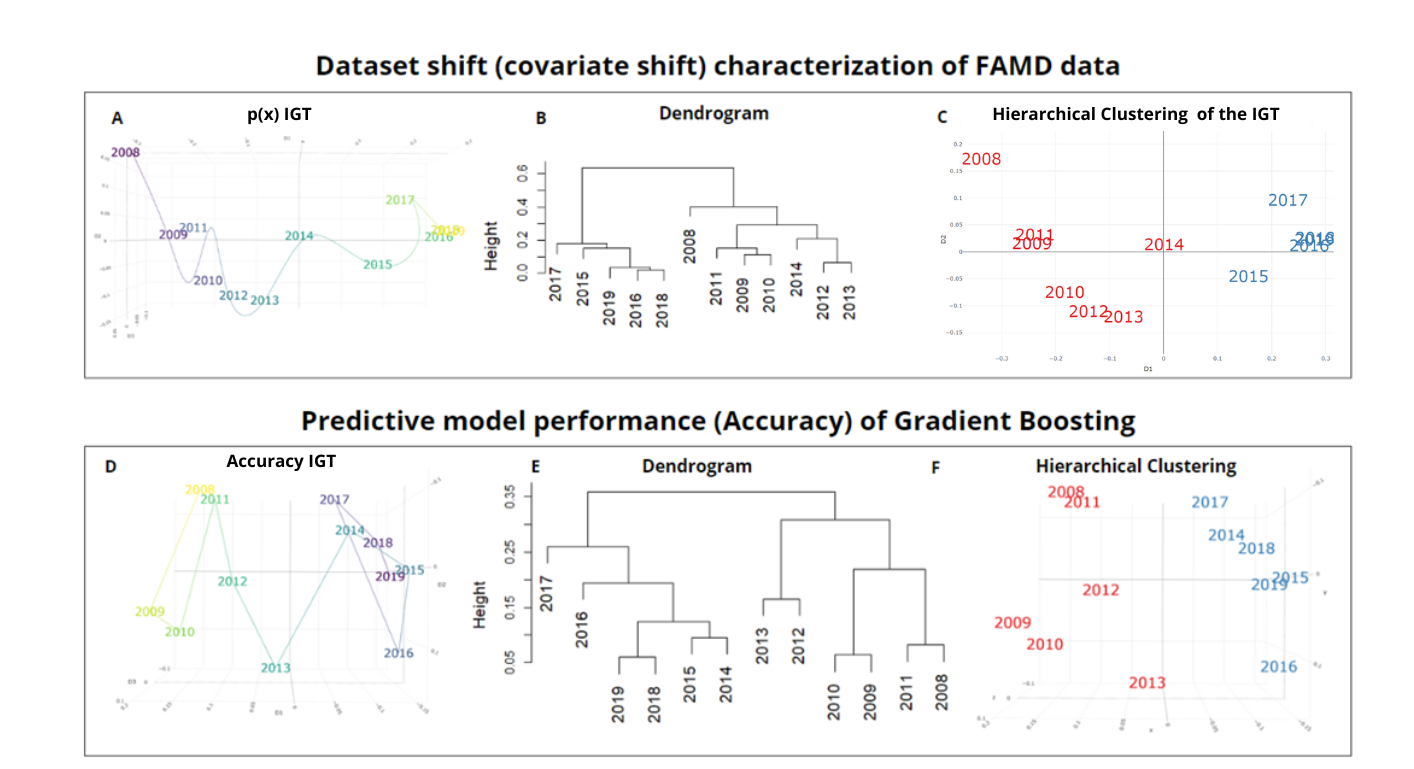


**Figure S10: Exploratory results of comparing dataset shift characterization of FAMD data covariate shift and Gradient Boosting model evaluation matrices of Accuracy. A** Information Geometric Temporal (IGT) of the initial 3 dimensions of the FAMD after applying a Kernel Density Estimation. **B** Dendrogram depicting the hierarchical clustering of the IGT for the initial three dimensions derived from the Factor Analysis of Mixed Data (FAMD), following the application of Kernel Density Estimation. **C** Representation of the hierarchical clustering of the IGT from the FAMD following the application of Kernel Density Estimation. **D** IGT of the evaluation matrix for the Accuracy metric of the Gradient Boosting model**.** **E** Dendrogram depicting the hierarchical clustering of the IGT for the evaluation matrix for the Accuracy metric on the Gradient Boosting model. **F** Representation of the hierarchical clustering of the IGT from the evaluation matrix for the Accuracy metric on the Gradient Boosting model

**Figure S9: Exploratory results of comparing dataset shift characterization of FAMD data covariate shift and Random Forest model evaluation matrices of Precision. A** Information Geometric Temporal (IGT) of the initial 3 dimensions of the FAMD after applying a Kernel Density Estimation. **B** Dendrogram depicting the hierarchical clustering of the IGT for the initial three dimensions derived from the Factor Analysis of Mixed Data (FAMD), following the application of Kernel Density Estimation. **C** Representation of the hierarchical clustering of the IGT from the FAMD following the application of Kernel Density Estimation. **D** IGT of the evaluation matrix for the Precision metric of the Random Forest model**.** **E** Dendrogram depicting the hierarchical clustering of the IGT for the evaluation matrix for the Precision metric on the Random Forest model. **F** Representation of the hierarchical clustering of the IGT from the evaluation matrix for the Precision metric on the Random Forest model


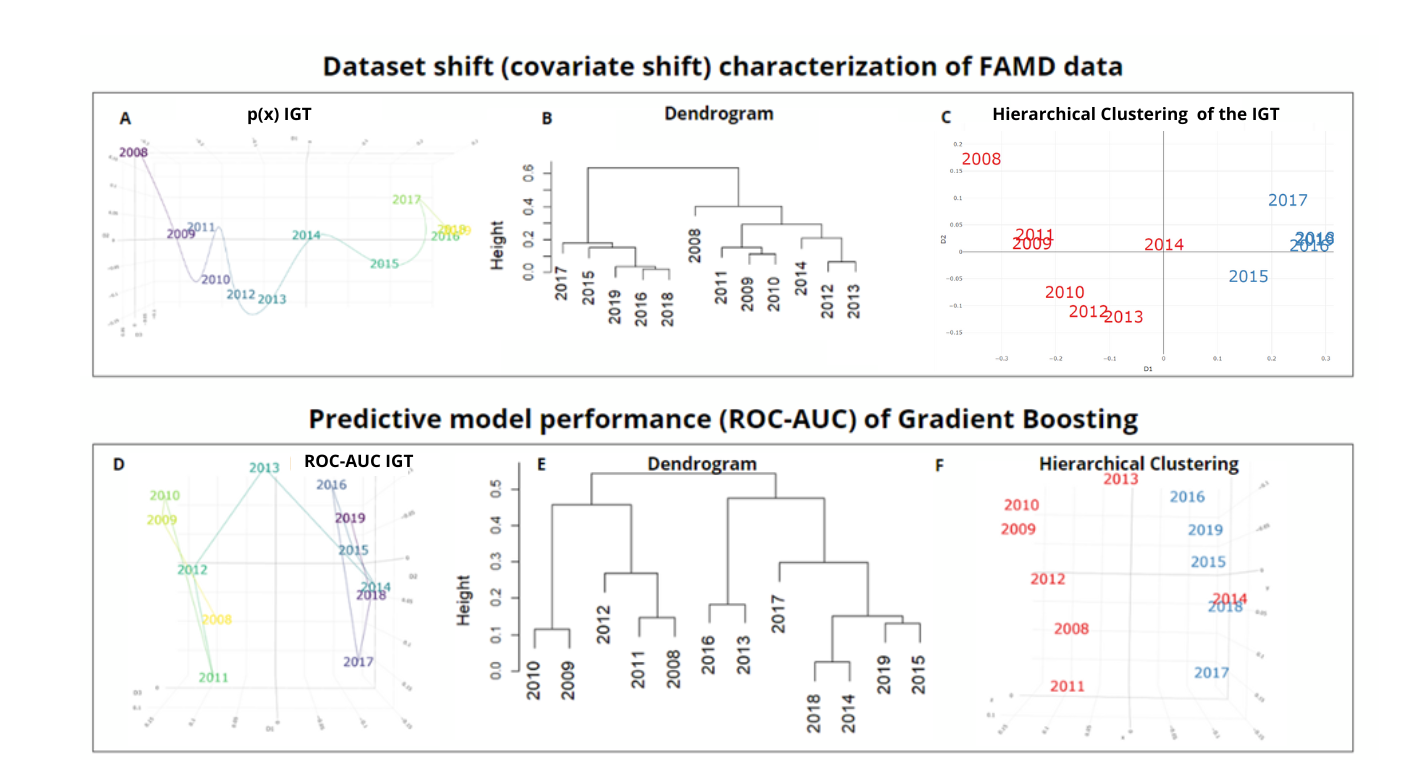

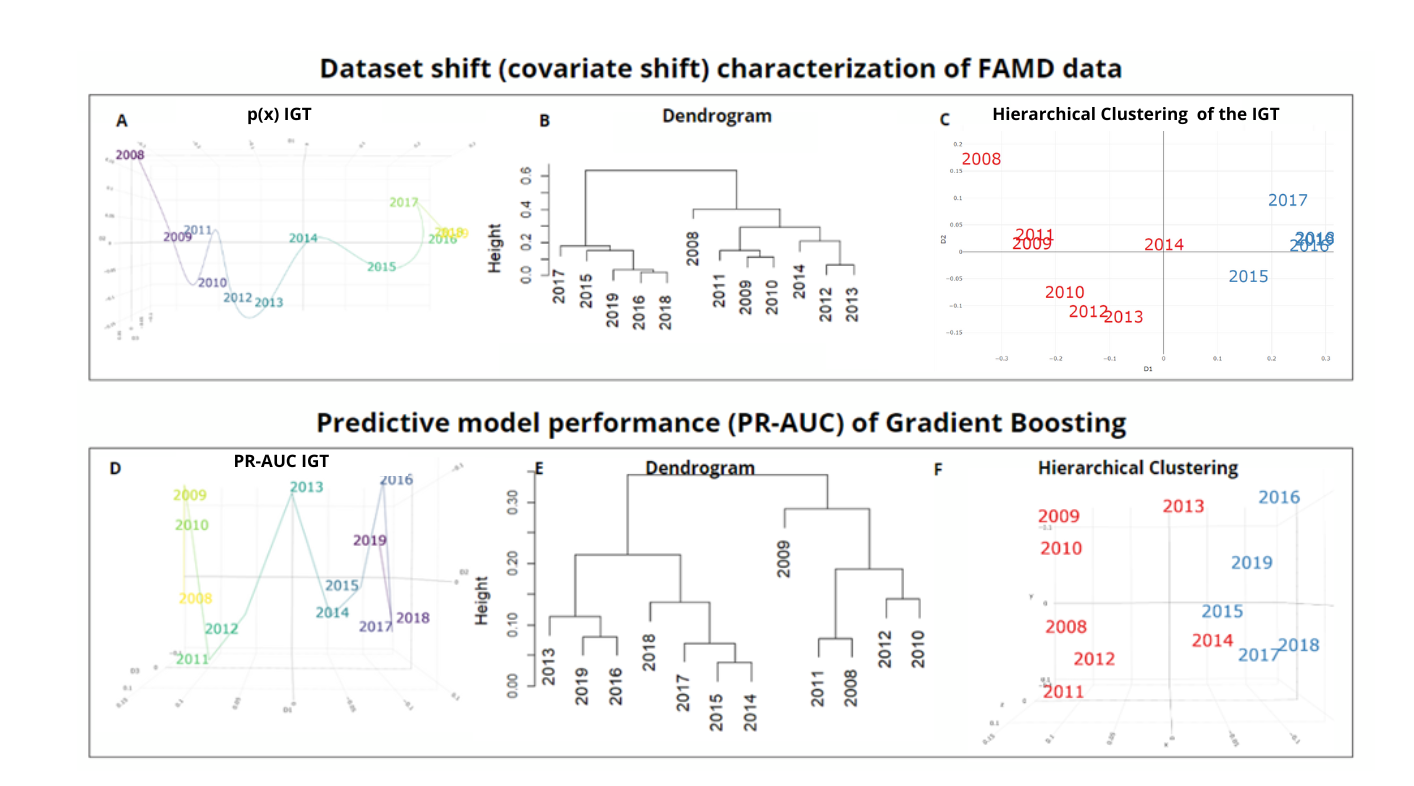


**Figure S11: Exploratory results of comparing dataset shift characterization of FAMD data covariate shift and Gradient Boosting model evaluation matrices of PR-AUC. A** Information Geometric Temporal (IGT) of the initial 3 dimensions of the FAMD after applying a Kernel Density Estimation. **B** Dendrogram depicting the hierarchical clustering of the IGT for the initial three dimensions derived from the Factor Analysis of Mixed Data (FAMD), following the application of Kernel Density Estimation. **C** Representation of the hierarchical clustering of the IGT from the FAMD following the application of Kernel Density Estimation. **D** IGT of the evaluation matrix for the PR-AUC metric of the Gradient Boosting model**.** **E** Dendrogram depicting the hierarchical clustering of the IGT for the evaluation matrix for the PR-AUC metric on the Gradient Boosting model. **F** Representation of the hierarchical clustering of the IGT from the evaluation matrix for the PR-AUC metric on the Gradient Boosting model


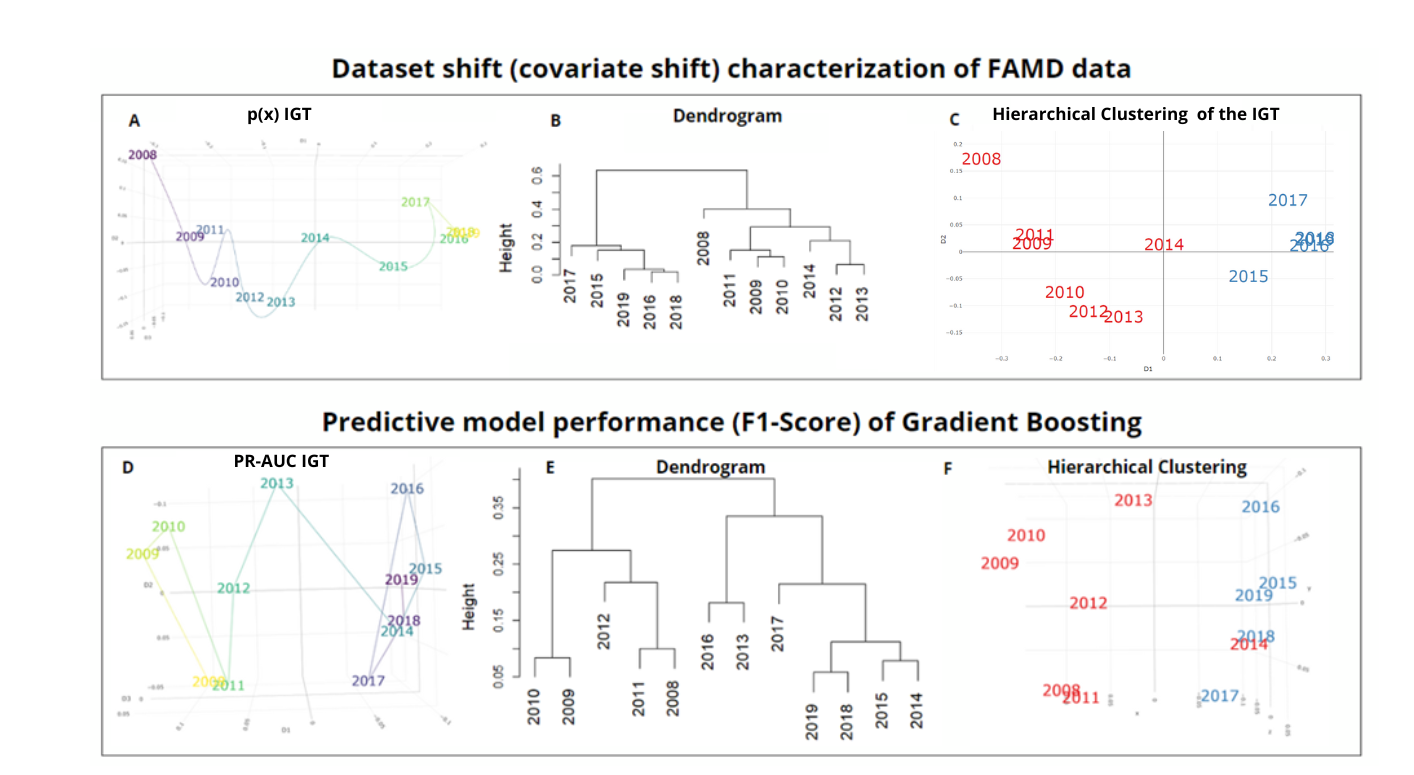


**Figure S13: Exploratory results of comparing dataset shift characterization of FAMD data covariate shift and Gradient Boosting model evaluation matrices of F1-Score. A** Information Geometric Temporal (IGT) of the initial 3 dimensions of the FAMD after applying a Kernel Density Estimation. **B** Dendrogram depicting the hierarchical clustering of the IGT for the initial three dimensions derived from the Factor Analysis of Mixed Data (FAMD), following the application of Kernel Density Estimation. **C** Representation of the hierarchical clustering of the IGT from the FAMD following the application of Kernel Density Estimation. **D** IGT of the evaluation matrix for the F1-Score metric of the Gradient Boosting model**.** **E** Dendrogram depicting the hierarchical clustering of the IGT for the evaluation matrix for the F1-Score metric on the Gradient Boosting model. **F** Representation of the hierarchical clustering of the IGT from the evaluation matrix for the F1-Score metric on the Gradient Boosting model

**Figure S12: Exploratory results of comparing dataset shift characterization of FAMD data covariate shift and Gradient Boosting model evaluation matrices of ROC-AUC. A** Information Geometric Temporal (IGT) of the initial 3 dimensions of the FAMD after applying a Kernel Density Estimation. **B** Dendrogram depicting the hierarchical clustering of the IGT for the initial three dimensions derived from the Factor Analysis of Mixed Data (FAMD), following the application of Kernel Density Estimation. **C** Representation of the hierarchical clustering of the IGT from the FAMD following the application of Kernel Density Estimation. **D** IGT of the evaluation matrix for the ROC-AUC metric of the Gradient Boosting model**.** **E** Dendrogram depicting the hierarchical clustering of the IGT for the evaluation matrix for the ROC-AUC metric on the Gradient Boosting model. **F** Representation of the hierarchical clustering of the IGT from the evaluation matrix for the ROC-AUC metric on the Gradient Boosting model


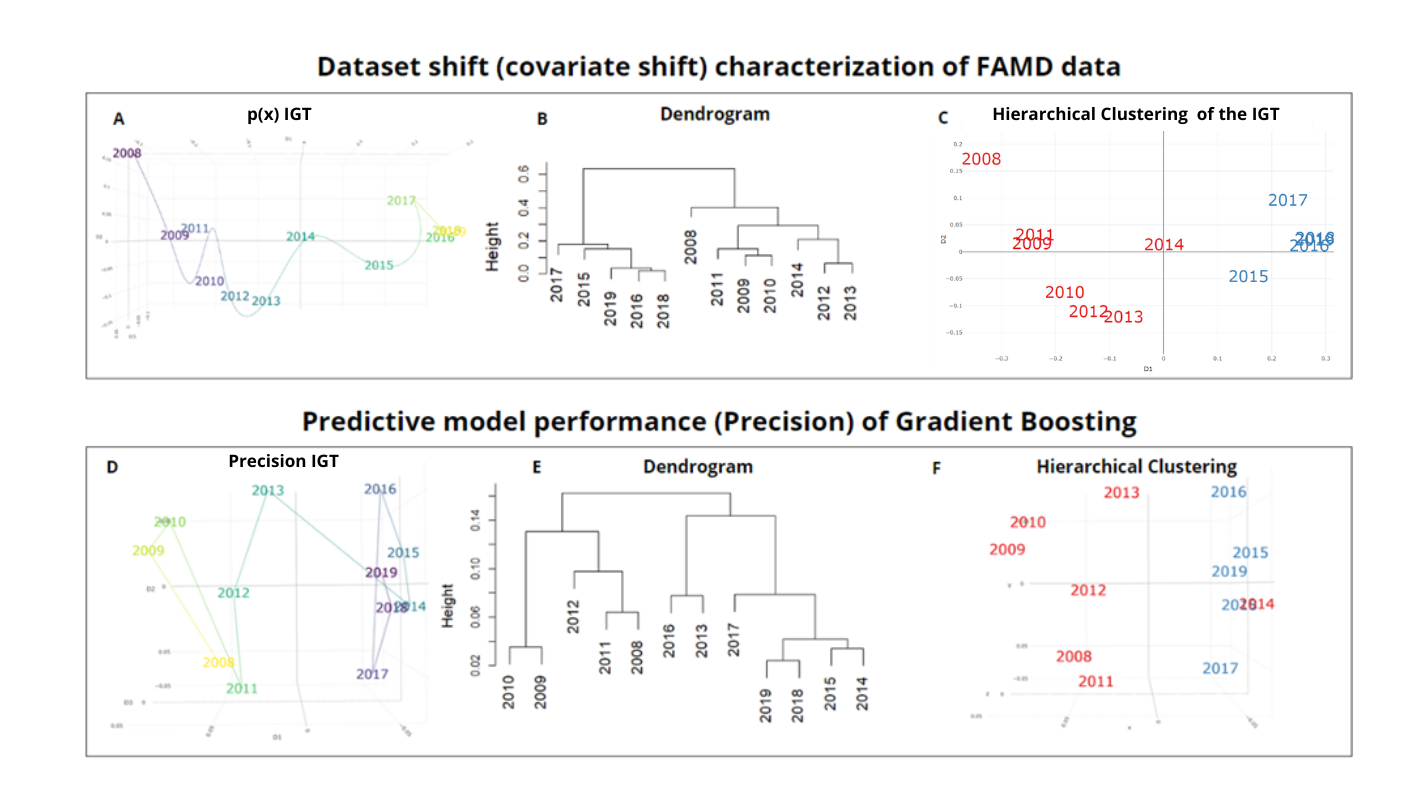


**Figure S14: Exploratory results of comparing dataset shift characterization of FAMD data covariate shift and Gradient Boosting model evaluation matrices of Precision. A** Information Geometric Temporal (IGT) of the initial 3 dimensions of the FAMD after applying a Kernel Density Estimation. **B** Dendrogram depicting the hierarchical clustering of the IGT for the initial three dimensions derived from the Factor Analysis of Mixed Data (FAMD), following the application of Kernel Density Estimation. **C** Representation of the hierarchical clustering of the IGT from the FAMD following the application of Kernel Density Estimation. **D** IGT of the evaluation matrix for the Precision metric of the Gradient Boosting model**.** **E** Dendrogram depicting the hierarchical clustering of the IGT for the evaluation matrix for the Precision metric on the Gradient Boosting model. **F** Representation of the hierarchical clustering of the IGT from the evaluation matrix for the Precision metric on the Gradient Boosting model


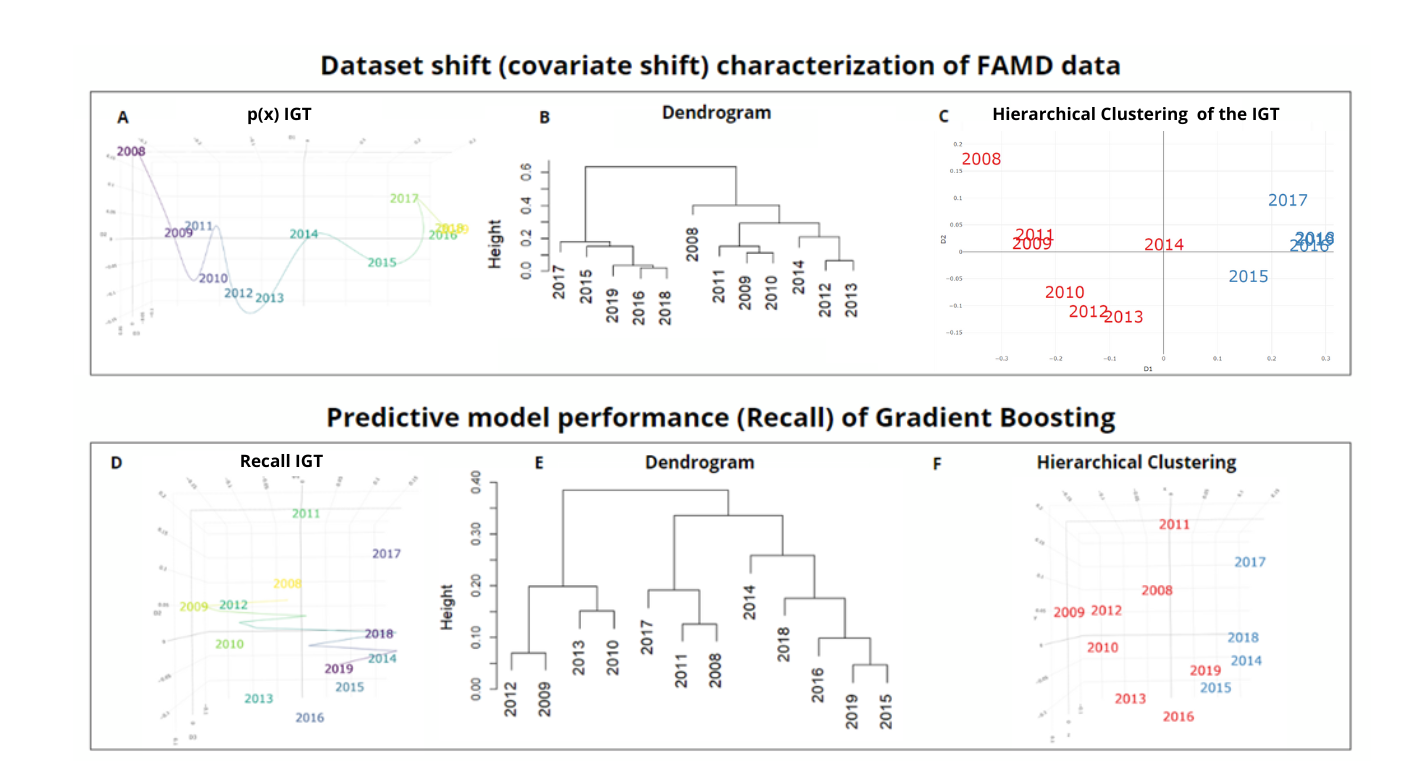


**Figure S15: Exploratory results of comparing dataset shift characterization of FAMD data covariate shift and Gradient Boosting model evaluation matrices of Recall. A** Information Geometric Temporal (IGT) of the initial 3 dimensions of the FAMD after applying a Kernel Density Estimation. **B** Dendrogram depicting the hierarchical clustering of the IGT for the initial three dimensions derived from the Factor Analysis of Mixed Data (FAMD), following the application of Kernel Density Estimation. **C** Representation of the hierarchical clustering of the IGT from the FAMD following the application of Kernel Density Estimation. **D** IGT of the evaluation matrix for the Recall metric of the Gradient Boosting model**.** **E** Dendrogram depicting the hierarchical clustering of the IGT for the evaluation matrix for the Recall metric on the Gradient Boosting model. **F** Representation of the hierarchical clustering of the IGT from the evaluation matrix for the Recall metric on the Gradient Boosting model

***Figure S16:
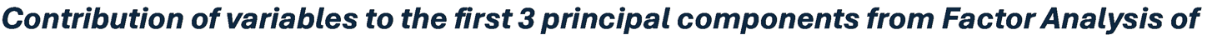
***


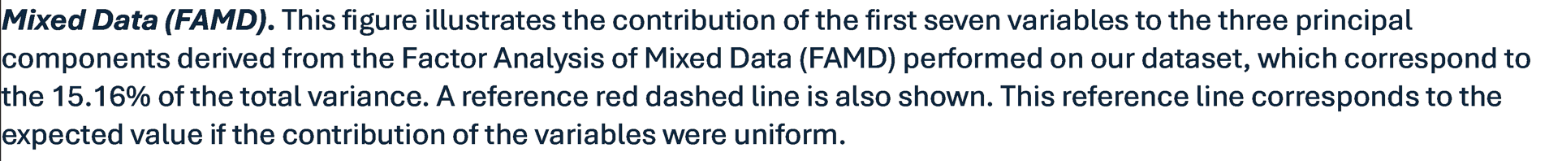


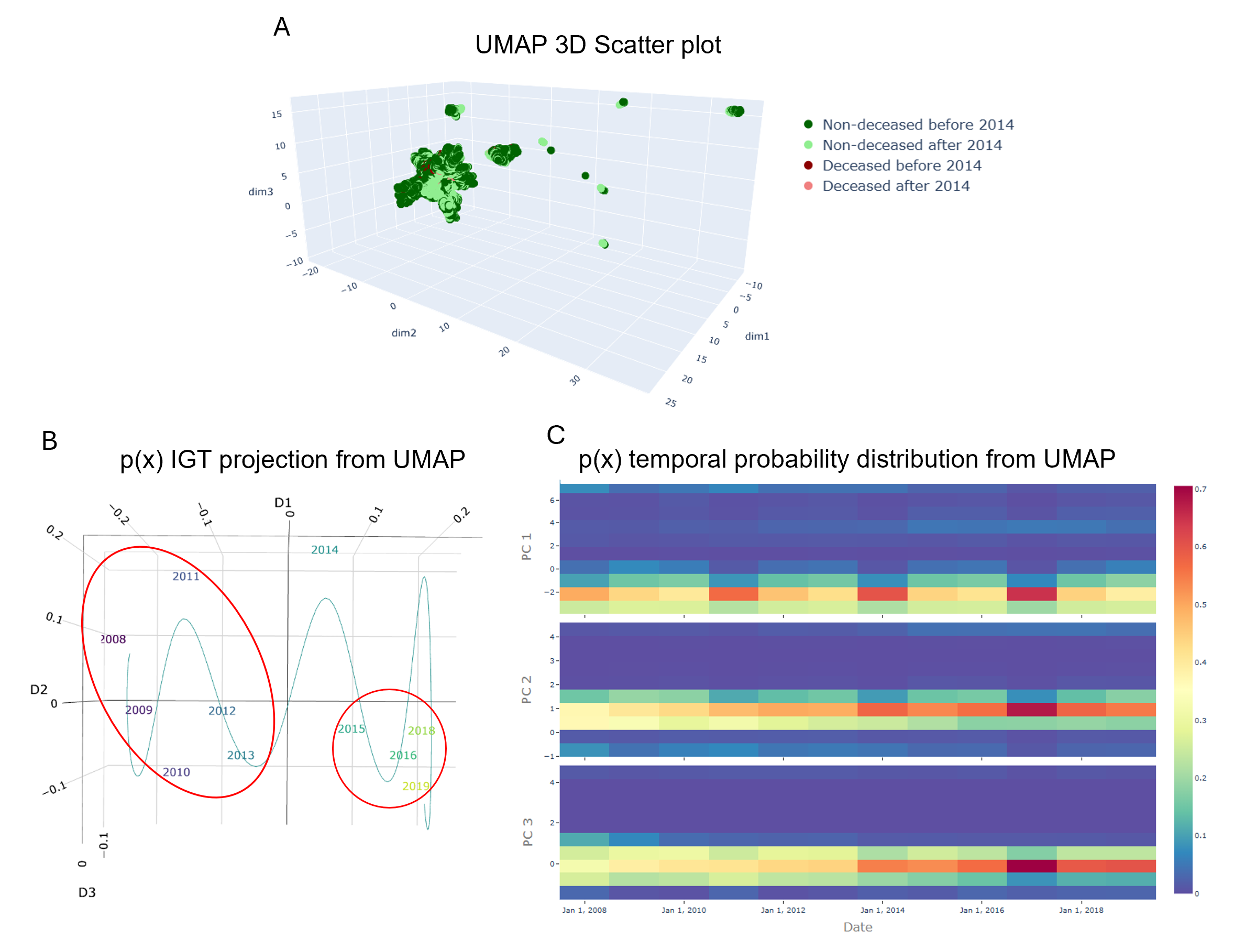


***Figure S17: Results of the multivariate dataset shift unsupervised characterization with UMAP.* A** *3D Scatter plot of of the studied MIMIC-IV dataset after performing dimensionality reduction with UMAP. The data is divided by classes (green and red) and time (dark and light colors).* **B** *IGT projection plot of the covariates after performing UMAP dimensionality reduction. Temporal subgroups are highlighted by red circles.* **C** *Heatmap showing the probability distribution of covariates over time across the three dimensions obtained from UMAP dimensionality reduction.*

**Table S8:** Results of the Fisher test after adjusting the p-values with False Discovery Rate (FDR) to compare two clustering methods for the Random Forest models: hierarchical clustering and K-means, both for k=2. Clustering was applied to the IGT obtained from the metrics of the Random Forest model and the IGT from the 3 principal dimensions of the FAMD data after applying the KDE. This comparison aimed to test the hypothesis of independence of both clusters (alpha = .05).

| Metric | p-value for hierarchical clustering | p-value for K-means |
| --- | --- | --- |
| ROC-AUC | .028* | .028* |
| PR-AUC | .028* | .028* |
| Precision | .028* | .028* |
| Recall | .028* | .028* |
| Accuracy | .028* | .028* |
| F1-Score | .028* | .028* |

* Independence is statistically significant (alpha = .05)

**Table S9:** Results of the Fisher test after adjusting the p-values with False Discovery Rate (FDR) to compare two clustering methods for the Gradient Boosting model: hierarchical clustering and K-means, both for k=2. Clustering was applied to the IGT obtained from the metrics of the Gradient Boosting model and the IGT from the 3 principal dimensions of the FAMD data after applying the KDE. This comparison aimed to test the hypothesis of independence of both clusters (alpha = .05).

| Metric | p-value for hierarchical clustering | p-value for K-means |
| --- | --- | --- |
| ROC-AUC | .028* | .028* |
| PR-AUC | .028* | .028* |
| Precision | .028* | .028* |
| Recall | .08 | .028* |
| Accuracy | .028* | .028* |
| F1-Score | .028* | .028* |

* Independence is statistically significant (alpha = .05)
